# Enhanced Expression of Mitochondrial Magmas Protein in Ovarian Carcinomas: Magmas Inhibition Facilitates Antitumour Effects, Signifying a Novel Approach for Ovarian Cancer Treatment

**DOI:** 10.3390/cells14090655

**Published:** 2025-04-29

**Authors:** Ali Raza, Ashfaqul Hoque, Rodney Luwor, Ruth M. Escalona, Jason Kelly, Revati Sharma, Fadi Charchar, Simon Chu, Mary K. Short, Paul T. Jubinsky, George Kannourakis, Nuzhat Ahmed

**Affiliations:** 1 Fiona Elsey Cancer Research Institute, Ballarat, VIC 3353, Australia; ali@fecri.org.au (A.R.); rodney@fecri.org.au (R.L.); ruth@fecri.org.au (R.M.E.); jason@fecri.org.au (J.K.); revati@fecri.org.au (R.S.); george@fecri.org.au (G.K.); 2 Institute of Innovation, Science and Sustainability, Federation University Australia, Health Innovation and Transformation Center, Mount Helen Campus, Ballarat, VIC 3050, Australia; f.charchar@federation.edu.au; 3 St Vincent’s Institute of Medical Research, Fitzroy, VIC 3065, Australia; ahoque@svi.edu.au; 4 Department of Surgery, Faculty of Medicine, Dentistry and Health Sciences, Royal Melbourne Hospital, Melbourne, VIC 3050, Australia; 5 Centre for Endocrinology and Reproductive Health, Hudson Institute of Medical Research, Clayton, VIC 3168, Australia; simon.chu@hudson.org.au; 6 Department of Molecular & Translational Science, Faculty of Medicine, Nursing and Health Sciences, School of Clinical Sciences at Monash Health, Monash University, Clayton, VIC 3168, Australia; 7 Developmental and Molecular Biology, Albert Einstein College of Medicine, New York, NY 10461, USA; mshortstop@frontier.com (M.K.S.); pajubins@montefiore.org (P.T.J.); 8 Department of Obstetrics and Gynaecology, Faculty of Medicine, Dentistry and Health Sciences, Royal Women’s Hospital, University of Melbourne, Parkville, VIC 3050, Australia; 9 Department of Surgery, Faculty of Medicine, Dentistry and Health Sciences, St Vincent Hospital, University of Melbourne, Fitzroy, VIC 3065, Australia

**Keywords:** ovarian cancer, Magmas, mitochondria, BT#9, reactive oxygen species, mitochondrial respiration, tumour growth, peritoneal metastasis

## Abstract

Mitochondrial-associated granulocyte macrophage colony-stimulating factor (Magmas) is a unique protein located in the inner membrane of mitochondria, with an active role in scavenging reactive oxygen species (ROS) in cellular systems. Ovarian cancer (OC), one of the deadliest gynaecological cancers, is characterised by genomic instability, affected by ROS production in the tumour microenvironment. This manuscript discusses the role of Magmas and efficacy of its novel small molecule inhibitor BT#9 in OC progression, metastasis, and chemoresistance. Magmas expression levels were significantly elevated in high-grade human OC compared to benign tumours by immunohistochemistry. The inhibition of Magmas by BT#9 enhanced ROS production and reduced mitochondrial membrane permeability, basal respiration, mitochondrial ATP production, and cellular functions, such as the proliferation and migration of OC cell lines in vitro. Oral administration of BT#9 in vivo significantly reduced tumour growth and spread and enhanced the survival of mice without having any effect on the peritoneal organs. These data suggest that Magmas is functionally important for OC growth and spread by affecting ROS levels and that the inhibition of Magmas activity by BT#9 may provide novel clinical benefits for patients with this malignancy.

## 1. Introduction

Ovarian cancer (OC) has been classified as the eighth most common cancer amongst gynaecological cancers in women worldwide [1], and in the USA, it is the second most common gynaecological cancer (https://www.cdc.gov/ovarian-cancer/statistics/index.html, accessed on 14 March 2025). The cancer is characterised by a poor prognosis with a ~45% five-year survival rate, which has not changed for the last two decades [2]. However, if the cancer is diagnosed when it is confined to the ovaries, International Federation of Obstetrics and Gynaecology (FIGO) stage I, the survival rate is 90%, and the majority of women survive long term with the current treatment approaches. Nonetheless, this survival rate decreases drastically as the cancer disseminates beyond the ovaries in the pelvic region (FIGO stages II and III) and the abdominal organs (FIGO stages III and IV). The low survival rate for OC patients is related mainly to the lack of specific early-stage screening tests and non-specific symptoms, which render the disease easily missed at the early stages, resulting in an advanced-stage diagnosis [3].

Ovarian cancer includes all malignant tumours arising in the ovaries, which can be of epithelial, germ cell, or stromal origin [4]. However, approximately 90% of OCs are of epithelial origin, with 80% of those being high-grade serous ovarian carcinomas (HGSOCs) [4,5]. These cancers manifest a loss of the functional p53 gene due to mutations, as well as other oncogenic pathways, including phosphatidylinositol 3 kinase (PI3K), retinoblastoma protein, sarcoma viral oncogene pathway, Notch, Hedgehog signalling pathways, etc. [5,6]. Recent genome-wide association and differentially expressed gene studies have identified several other genes that are mutated or differentially expressed in HGSOCs compared to benign tumours or normal ovaries [7,8,9]. Based on these studies, several clinical trials targeting the identified genes with or without standard chemotherapies are in progress or completed, but none have shown significant improvements [10]. Hence, new strategies are required to obtain more favourable outcomes.

To design better therapeutic options for OC patients, it is important to understand the unique tumour microenvironment (TME), which promotes the growth of cancer cells. The TME of OC is unique as it encompasses both solid tumours as well as floating aggregates of tumour cells (spheroids), which are shed from the originating ovarian surface epithelium or the epithelium of fallopian tubes [11]. These tumours grow and disseminate within the abdominal cavity. Adding to that scenario, ascites (tumour fluid) in small volumes or in several litres is present in the abdomen of OC patients, which creates shear stress on the tumour cells, which enhances tumour cell plasticity and promotes metastasis [12,13]. In that context, the mere abundance of tumour load within the abdomen creates a hostile TME with advancing hypoxia and low levels of essential nutrients, including glucose [14,15]. This unique alteration in TME promotes the production of harmful ROS, prompting the cancer cells to adapt to an altered TME with reprogrammed metabolism for survival [15].

Mitochondria are the organelles that integrate nearly all cellular processes, including glucose and lipid metabolism, protein synthesis and degradation, apoptosis, and transcription [16]. Intracellular mitochondria continuously undergo division to form a dynamic mitochondrial network which preserves the cell’s integrity and provides flexibility to adapt to the changed cellular microenvironment by altering cellular metabolism [17]. Mitochondrial irregularities and dysfunction have been demonstrated in cancer, including OC [18,19]. OC tumours demonstrated a 3–8-fold increased mitochondrial content and altered mitochondria morphology, with a reduction in mitochondrial cristae width and junction diameter [20,21,22]. In addition, mutations in the mitochondrial DNA of OC cells have been observed [21]. These findings suggest that altered mitochondrial function in OC cells may induce or maintain the malignant phenotype.

Magmas is an essential nuclear-encoded highly conserved mitochondrial protein present in all eukaryotic cells. It was identified as an early granulocyte macrophage colony-stimulating factor (GM-CSF)-induced gene when a growth factor-dependent cell line was cultured in IL-3 [23]. Antisense to Magmas inhibited the proliferation of these cells cultured in GM-CSF but not IL-3 [23,24,25]. Magmas protein is an ortholog of mitochondrial pre-sequence translocated-associated motor subunit (PAM)16 translocase of the inner membrane subunit (TIM)16 in yeast cells and acts as a co-chaperone of mitochondrial heat shock protein 70 (mtHsp70) [26,27]. Magmas is associated with the inner mitochondrial membrane in humans and forms a stable subcomplex with heat shock protein 70 (Hsp70), PAM17, and the mitochondrial inner membrane J-proteins PAM18 (DNAJC19), and it is needed for the formation of an mtHsp70–TIM44 complex, which is then bound to the TIM23 complex [24,27,28,29]. The TIM23 complex bound to Magmas regulates the import process by inhibiting the human mtHsp70 ATPase stimulatory activity and controls the import of various nuclear-encoded mitochondrial proteins into the mitochondrial matrix that are important in maintaining ROS homeostasis, potentially through binding to complex IV of the electron transport chain (ETC) [24,30,31,32]. The deletion of PAM16 in yeast cells was found to be lethal for their survival, thus proving its important role in yeast cell survival [33]. In humans, Magmas was observed to be upregulated in a PGMD1 pituitary adenoma cell line. These studies showed that the reduced expression of Magmas resulted in a decreased proliferation of PGMD1 cells [23]. Magmas have been reported to be overexpressed in adrenocorticotrophic hormone (ACTH)-secreting pituitary adenomas [34]. The silencing of Magmas in two murine pituitary cell lines overexpressing Magmas resulted in growth arrest at the Go/G1 phase [34]. An enhanced expression of Magmas has been shown to provide tolerance towards oxidative stress by enhancing the activities of antioxidant enzymes, and the activity of electron transport chain complexes causing reduced intracellular production of ROS, thus protecting cells from oxidative damage [35]. The expression of Magmas was also shown to induce a protective role against the apoptotic stimulus of the protein kinase C (PKC) inhibitor staurosporine in the murine ACTH-secreting pituitary adenoma cell line and the rat pituitary adenoma cell line-secreting growth hormone/prolactin (GH/PRL) [36]. An increased expression of Magmas has been noted in prostate cancer [37], glioblastomas [38], triple-negative breast cancers [39], neuroendocrine prostate cancers [40], and recently, in ovarian cancer [41]. In addition, Magmas mutation has been linked to skeletal dysplasia, indicating a specific role of this mitochondrial protein in bone formation [42]. 

A small molecule inhibitor of Magmas, BT#9, was designed to bind to Magmas and block its activity, as evidenced by its inhibitory effect on the proliferation of *S. cerevisiae* containing different mutants of Magmas plasmids [43]. In preclinical models of brain tumours, BT#9 has shown good efficacy against brain tumours by crossing the blood–brain barrier [38]. Studies with BT#9 on osteosacrcoma, leukemia, lymphoma, prostate cancer, and brain tumour cell lines showed a 6- to 32-fold more potent inhibition than primary hematopoietic cells and human umbilical vein endothelial cells (HUVECs) [44]. In addition, BT#9 treatment reduced the respiratory function of glioma cells, reinforcing the role that Magmas has as a potent ROS regulator [38]. We have previously shown that BT#9 has greater sensitivity towards platinum-resistant OC cells compared to control platinum-sensitive cells [41]. These results suggest a greater benefit of using BT#9 in clinical scenarios of OC patients compared to standard chemotherapies currently in practice.

In this study, we extensively analysed the expression of Magmas protein in different stages and grades of ovarian cancer and systematically studied how BT#9 affects, mitochondrial phosphorylation, mitochondrial membrane permeability, ROS production, superoxide dismutase (SOD) activity, proliferation, migration, chemosensitivity in vitro, tumour progression and overall survival in a nude mouse model. Our data also show that BT#9 is much less effective in killing normal fallopian tube-derived FT282 cell lines compared to OC cell lines. These data are consistent with the data in a mouse model where oral gavage of BT#9 for two weeks failed to show any histological damage to the abdominal organs, while at the same time, in a separate experiment, reduced significantly OC cells induced tumour burden and prevented the infiltration of OC cells to abdominal organs in mice, suggesting a greater benefit of using BT#9 in clinical scenarios of OC patients compared to the standard chemotherapies currently in practice.

## 2. Materials and Methods

### 2.1. Chemicals and Reagents

BT#9 was synthesised as described previously [43] and was dissolved in captisol (sulfobutylether-β-cyclodextrin), a modified cyclodextrin used to facilitate the solubility, stability, and bioavailability of insoluble ingredients. Captisol has a good clinical safety record and is currently used as a vehicle for the administration of multiple Food and Drug Administration (FDA)-approved prescription drugs. Captisol on its own had no detectable inhibitory effects on cultured cells or in mice.

### 2.2. Collection of Ovarian Tumours from Patients

#### 2.2.1. Ethics Approval

The human ovarian cancer tumours used in this study were from surgically resected specimens donated by patients with consent, diagnosed with epithelial ovarian serous cancer or benign tumours of the same origin. This study was performed under Human Ethics applications endorsed by The Research Ethics Committee of Royal Women’s Hospital (RWH) (HEW#09/09) and Victorian Cancer Biobank (VCB-17018), Melbourne, Australia. This study was also sanctioned for Fiona Elsey Cancer Research Tissue Bank by Ballarat Health Services (Project ID: 37521).

#### 2.2.2. Collection of Ovarian Tumours

Epithelial serous ovarian tumours were obtained from patients diagnosed with ovarian cancer and admitted for surgery at RWH or from VCB, Melbourne, Australia. Benign tumours were accrued from women undertaking abdominal hysterectomy or bilateral salpingo-oophorectomy due to prior medical history. Tissues were fixed in 4% paraformaldehyde at the time of collection. Patient information, such as tumour grade and stage of tumours, was obtained from RWH or VCB pathology reports. Patients who participated in this study were chemo-naïve and did not go through any form of treatment. Clinical information on patients who participated in this study is described in Supplementary Table S1.

### 2.3. Immunohistochemical Analyses of Magmas in Ovarian Tumours

Human ovarian tumours were subcontracted to the Anatomical Pathology Laboratory Services, The Royal Children’s Hospital, Melbourne, Australia. The immunohistochemistry analysis was performed as described previously [45]. In brief, paraffin-embedded tumours sectioned at 4 μm thickness were incubated with anti-Magmas antibody (1:1000) [25] followed by secondary antibody, anti-rabbit IgG (H+L) horse radish peroxidase (HRP) (Bio-Rad, Sydney, Australia catalogue #1706515), and reagents in an OptiView 3,3′-diaminobenzidine (DAB) immunohistochemical (IHC) detection kit (Ventana Medical Systems, Inc., Tucson, AZ, USA). The samples were processed on a Ventana Benchmark Immunostainer (Ventana Medical Systems, Inc., Tuscon, AZ, USA). Negative controls included incubating tumour sections in secondary antibody only, without the addition of a primary antibody. Human placental and tonsil tissue sections were used on each slide as positive controls. Microscopically, the sections were assessed for positive DAB staining. Imaging was obtained via the EVOS FL Auto 2 cell imaging system (Thermo Fisher Scientific, Melbourne, Australia) and then analysed using ImageJ software, version 1.54g (Bethesda, MD, USA). The parameters analysed were the mean intensity of the DAB staining of the deconvoluted image of the IHC images. The mean intensity data of the slides obtained from different ovarian cancer patients were compared. The results were plotted on a bar graph using PRISM software, version 10.4.1 for Windows (Boston, MA, USA), then analysed and sorted according to FIGO stages and Silverberg grades.

### 2.4. Ovarian Cancer Cell Lines

Established human OC cell lines, HEY, OVCAR5, A2780, OAW28, SKOV3, OV90, COV318, COV504, COV362, OVCAR3, and ES2, were obtained from the laboratory of Dr. Carmela Ricciardelli, Adelaide Medical School, Adelaide University, Australia. The normal fallopian epithelium-derived cell line, FT282, immortalised by the transfection of vectors expressing human telomerase reverse transcriptase (pBABE-hygro-TERT) and mutant p53R175H (pLenti6/V5-TP53R175H), was a kind gift from Prof. David Bowtell (Peter MacCallum Cancer Centre, Parkville, Australia) [46]. The OVCAR5 cell line initially collected from the ascites of an untreated, advanced-stage ovarian cancer patient, was later found to be of gastric origin by gene expression analysis [47]. However, a paper has confirmed this cell line as of ovarian origin by short tandem repeat (STR) sequencing [48]. The ES2 cell line, originally identified to have ovarian clear cell carcinoma characteristics, was recently identified to have ovarian high-grade serous characteristics by histopathological analysis [49]. Information on all cell lines including origin, disease progression, and growth conditions is provided in Table 1. All cells were maintained at 37 °C in 5% CO_2_ humidity and were passaged at least twice a week once they reached a confluence of 65–80%.

### 2.5. Immunofluorescence (IF) on Ovarian Cancer Cell Lines

OC cell lines were seeded at a 1 × 10^5^ concentration on sterile 10.5 × 22 mm coverslips placed in 6-well plates and allowed to grow overnight in appropriate media. Cells were washed and fixed in 4% paraformaldehyde and treated with 0.01% Triton-X for 30 min, followed by phosphate-buffered saline with Tween 20 (PBST) washes. After blocking with 5% bovine serum albumin (BSA), the cells were incubated with Magmas antibody (1:1000) [23] for 1 h followed by Alexa Flour 555 antibody for 1 h and then stained with 4′,6-diamidino-2-phenylindole (DAPI 1:1000, Invitrogen, Melbourne, Australia). The coverslips were mounted on slides using ProlongTM Diamond Antifade mountant (Thermo Fisher Scientific, Melbourne, Australia) and allowed to dry overnight in a cool, dark, and dry environment. The cells were then imaged using an Evos microscope FL Auto 2 cell imaging system (Thermo Fisher Scientific, Melbourne, Australia) using the DAPI and RFP filters. The settings for light and exposure were kept consistent across slides for comparison between cell lines.

### 2.6. Western Blot (WB)

For Western blot, 2 × 10^6^ cells were seeded in 25 cm^2^ flasks the night before the cells were collected for Western blot analysis. Western blot was performed on the OC cell lysates using sodium dodecyl-sulphate polyacrylamide gel electrophoresis (SDS-PAGE) by the methods described previously [53]. Total protein (40 μg) was separated on pre-cast SDS-PAGE gel (Bio-Rad, Sydney, Australia) and transferred to a polyvinylidene difluoride (PVDF) membrane. After blocking with 5% skimmed milk, the membranes were stained with Magmas antibody (1:1000), followed by treatment with anti-rabbit IgG (H+L) horse radish peroxidase (HRP) secondary conjugated antibody (Bio-Rad, Sydney, Australia). Protein bands were developed using the enhanced chemiluminescence reagents (Bio-Rad Laboratories, Melbourne, Australia) and visualised using the ChemiDoc imaging system (Bio-Rad, Australia). Using densitometric Image Lab 6.0 software (Bio-Rad, Sydney, Australia), the bands for each image were quantified.

### 2.7. Water-Soluble Tetrazolium Salts 1 (WST-1) Assay

The 3 × 10^4^ cells were seeded into 96-well plates overnight. Cell lines were treated with different concentrations of BT#9 for 24 h. The WST-1 assay (10%, Sigma-Aldrich, Melbourne, Australia) was performed as described in the manufacturer’s instructions. Absorbance was read at OD_450nm_ using the iMarkTM (Bio-Rad, Sydney, Australia). The results were plotted using PRISM software.

### 2.8. Active Caspase 3/7 and Propidium Iodide Assays

HEY and OVCAR5 cell lines at a density of 5 × 10^4^ cells were grown in 24-well plates (Corning Incorporated Costar, Corning, NY, USA) until 80% confluent. The cells grouped as control (normal growth medium RPMI without Phenol Red, Gibco, Melbourne, Australia) BT#9 concentrations of IC_50_ or IC_25_ were treated with Incucyte^®^ active caspase 3/7 (Sartorius, Gottingen, Germany) and propidium iodide (Biolegend, Perth, Australia) for 12 h. The images of the control and treated cells were obtained using the Evos microscope (Aperio Technologies, Vista, CA, USA). The GFP channel and RFP channel were used for caspase 3/7 activation propidium iodide staining. The experiments were carried out in 3 biological replicates where multiple images were taken from each well, and the fluorescence of the samples was measured as the median intensity of fluorescence using Image J Fiji software (Wayne Rasband National Institute of Health) and processed using package ImageJ2.

### 2.9. TMRM Assay for the Measurement of Mitochondrial Membrane Potential

Mitochondrial membrane potential was measured using the fluorescence probe tetramethylrhodamine methyl ester (TMRM; Invitrogen, Cat M20036) as per the manufacturer’s instructions. Briefly, 2.5 × 10^4^ cells were seeded into 200 μL, flat bottom, 96-well plates (Corning Life Science, Melbourne, Australia). After 6 or 9 h treatments, the cells were cultured in 20 nM TMRM for 30 min at 37 °C and 5% CO_2_. Carbonyl cyanide 3-chlorophenylhydrazone (CCCP) (50 μM) was added to one of the control wells to block the mitochondrial uptake of TMRM 10 min prior to the addition of TMRM. After TMRM incubation, the experimental wells containing the cells were washed in PBS and trypsinised for cell harvesting. The harvested cells were stained on ice with Fixable Viability Stain 700 (BD Horizon Cat 564997). A total of 7000 cells were analysed using a four-laser LSR Fortessa (BD Biosciences, Melbourne, Australia), with TMRM detected in the PE channel (Excitation 561 nm, Emission detector 586/15 nm). Dead cells were identified as positive for Fixable Viability Stain 700 (Excitation 640 nm, Emission detector 730/745 nm). FlowJo software (v. 10.10.0; FlowJo-Tree Star Inc. Ashland, OR, USA) was used to analyse flow cytometric data, calculate the median fluorescence intensity (MFI), and prepare fluorescence histograms.

### 2.10. DCFDA Assay to Measure ROS Production

For this assay, 5 × 10^4^ cells/well were seeded in 24-well plates for 12 h. The DCFDA assay kit comprises a cell-permeable redox-sensitive fluorescent probe that is oxidised by ROS and certain reactive nitrogen species (RNS) to yield the highly fluorescent product, 2,7-dichlorofluoroscein (DCF) (Abcam, Melbourne, Australia). The assay was carried out by immunofluorescence as described in the manufacturer’s instructions.

In brief, HEY or OVCAR5 cells grown to 80% confluency in 24 wells for 12 h were treated with IC_25_ or IC_50_ concentrations of BT#9 and/or tert-butyl hydrogen peroxide (TBHP, ROS-causing agent, 150 µM) for 12 h. After a sterile PBS wash, cells were incubated with diluted DCFDA in the dark at 37 °C in a 5% CO_2_ incubator for 45 min. The cells were imaged on the GFP channel using the Evos microscope. Using ImageJ software, the median fluorescence intensity of all the DCF-stained cells from different treatment groups was plotted using PRISM software.

### 2.11. Superoxide Dismutase Assay (SOD)

A superoxide dismutase (SOD) kit (Sigma-Aldrich, Sydney, Australia) measures the activity of the SOD enzyme, which regulates ROS production via catalysing superoxide anion (O_2_^−^) into hydrogen peroxide and molecular oxygen. This kit utilises the WST-1 solution, which measures the formation of tetrazolium salt after reduction with O_2_^−^. The rate of reduction in O_2_^−^ is linearly proportional to the activity of xanthine oxidase, which is inhibited by cellular SOD. The assay allows for determining the inhibition of SOD via the WST-1 assay.

HEY and OVCAR5 cell lines were grown at a density of 5 × 10^4^ cells/well in 24-well plates overnight and treated with IC_25_ or IC_50_ concentrations of BT#9 for 12 h. Following the protocol described in the instruction manual, both control and treated cells were set up with buffers along with the WST-1 working solution included in the assay kit. The plates were incubated for 20 min at a 37 °C in a CO_2_ incubator, and the absorbance was obtained using an iMARK microplate reader (Bio-Rad, Sydney, Australia). The inhibition of SOD was calculated as described in the manufacturer’s instructions and is expressed as SOD activity units/mL.

### 2.12. Cell Bioenergetic Assay

Cellular bioenergetic profiling of HEY and OVCAR5 cell lines in the presence and absence of BT#9 was assessed using the Mito stress assay kit (Agilent Technologies, Melbourne, Australia) on a Seahorse Extracellular Flux XFp Analyzer (Agilent Technologies) according to the manufacturer’s instructions. Briefly, the sensor cartridge was hydrated with calibrant overnight prior to cell seeding. Cells treated with either vehicle (captisol), IC_25_, or IC_50_ concentrations of BT#9-treated cells (3 × 10^4^) and were plated in 2 technical replicates across biological triplicates. Additionally, two wells were used containing growth media, which served as a background correction. Twelve hours post-seeding, the Mito stress test template was selected, and the utility plate along with the cartridge was loaded onto the machine for calibration. After instrument calibration, a miniplate with the vehicle and BT#9-treated cells was loaded onto the Seahorse Analyzer. The stressors used to assess electron transport complexes (ETCs) included Rotenone (complex 1 inhibitor), Antimycin A (complex 3 inhibitor), carbonyl cyanide-4-trifluoromethoxy phenylhydrazone (FCCP, which disrupts mitochondrial membrane potential), and oligomycin (complex V inhibitor). The stressors were sequentially injected into the wells over the course of the assay. Raw data were normalised against the total protein amount, measured using the Pierce BCA protein assay (Thermo Fisher Scientific, Melbourne, Australia). After completion of the assay, the data were analysed using the proprietary Agilent Wave software version 2.6.031. to calculate basal respiration, ATP-linked respiration, maximal and reserve capacities, and non-mitochondrial respiration.

### 2.13. Cell Proliferation and Migration Assays by xCELLigence

Real-time cell analyses (RTCAs) were conducted using the Agilent xCELLigence RTCA DP (dual-purpose) instrument. For proliferation assays, cells were grown to 80% confluence and serum starved overnight Gibco^®^Opti-MEM™ Media (Thermo-Fisher Scientific, Melbourne, Australia) for G0 synchronisation prior to the commencement of the experiment. Each E-plate (Agilent Technologies, 5469830001) was then equilibrated using 50 µL of complete media per well for 1 h prior to checking the background reading. Cells were then seeded at 1 × 10^4^ cells/140 µL/well of complete serum medium +/− BT#9 at IC_25_ and IC_50_ concentrations of BT#9 and corresponding untreated and captisol (1.32 µM) control cells.

For migration assays, a 16-well CIM plate (Agilent Technologies, #5665817001) was equilibrated with 30 µL pre-warmed serum-free media (Gibco^®^Opti-MEM™ Media (Thermo-Fisher Scientific, Melbourne, Australia) in the upper chamber, while lower chambers of the CIM plate were filled with 160 μL of complete serum medium and placed in a 37 °C incubator for 1 h to adjust the background reading. On the top well compartment, cells were then seeded at 4 × 10^4^ cells suspended/160 μL of serum-free medium +/− BT#9 at IC_25_ and IC_50_ concentrations and corresponding untreated and captisol controls. Impedance readings were taken every 15 s for the first 6 h and then 15 min for the experimental duration of both proliferation and migration assays. All RTCA assays were performed in duplicate for three independent experiments. The mean (±SEM) results were illustrated graphically using PRISM software.

### 2.14. Methyl Thiazol Tetrazolium (MTT) Assay

This assay was performed as described previously [50]. In brief, 3 × 10^4^ cells were seeded in 96-well plates overnight. OVCAR5 and HEY cell lines were treated for 48 h with different concentrations of paclitaxel (PTX) (EbeweR, SANDOZ, Novartis, Basel, Switzerland). This experiment was carried out first to obtain the right PTX IC_50_ amount for each cell line. Then, cell lines were treated for 48 h with different concentrations of BT#9 (control) or with PTX IC_50_ + different concentrations of BT#9 (PTX treated) for 48 h. The MTT assay involved replacing the culture media with 100 μL of thiazolyl blue tetrazolium (MTT) solution (Sigma-Aldrich) dissolved in PBS (0.5 mg/mL) (Sigma-Aldrich) and incubating cells for 2 h at 37 °C in 5% CO_2_. After 48 h, the MTT solution from the top of 96-well plates was discarded and replaced with 100 μL of dimethyl sulfoxide (DMSO, Sigma-Aldrich). Absorbance was read at OD_595nm_ using the CLARIOstar Plate Reader (BMG Labtech, Ortenberg, Germany). The data were analysed using MARS Data Analysis Computer Software, version 3.32 (BMG Labtech, Melbourne, Australia). Each plate contained four duplicate wells in each run for three different experiments, and the mean results were illustrated graphically using PRISM software. Linear regression analyses of control and treatment group slopes were used to determine IC_50_ amounts.

### 2.15. RNA Extraction and qRT-PCR

RNA was extracted from snap-frozen mouse xenograft sections stored in TRIzol^®^ reagent (Thermofisher Scientific, Melbourne, Australia) followed by the chloroform:phenol method as described previously [54]. RNA (500 ng) was reverse transcribed using the high-capacity cDNA Reverse Transcription Kit (Thermofisher Scientific, Melbourne, Australia). Quantitative real-time PCR on mouse xenografts was performed as described previously [53] using the QuantStudio™ 6 Real-Time PCR System (Thermo Fisher Scientific, Melbourne, Australia). Relative gene expression was calculated as 2−ΔΔCt using 18S as the endogenous reference gene and the average of the controls as the calibrator. All PCR reactions were performed in triplicate. The primer sequences and product size of Magmas gene is provided below: 

Gene symbol Accession no.    Primer Sequence (5′-3′)       Product size (bp)

Magmas   NM_001201479.1  Forward GCAGATTCTCAACGTGTCCA  188

               Reverse GCATCTGCCCTTTTTCTCTG

Data is presented as relative expression normalised to the housekeeping gene 18S.

### 2.16. Animal Experiments

#### Animal Ethics Statement

The animal experiments were carried out at the University of Melbourne, Parkville Animal Facility of Melbourne Bioresources Platform, medical building 181, Parkville, Victoria, Australia. The animal studies were approved by the animal ethics committee of Melbourne University, animal ethics study ID#1814509.3. The procedures for all the in vivo experiments were carried out following the strict guidelines directed by the National Health and Medical Research Council (NHMRC), Australia.

The experiments were performed as described previously [53]. Briefly, female Balb/c nu/nu mice (6–8 weeks old) were obtained from the Animal Resources Centre, Western Australia. HEY OC cell line (5 × 10^6^ cells/100 μL), a dose previously shown to produce significant tumour burden, was injected intraperitoneally (IP) into each mouse (n = 5/group) [55]. After 18 days, mice were treated daily with oral gavage of BT#9 (diluted in captisol, 30 mg/kg/body weight of mice). Mice were inspected every day, and tumour formation was monitored by palpation in addition to the overall body condition and body weight until the predetermined endpoint (determined by the ethics, which included tumour burden hampering mobility of mice, body weight loss greater than 15% of initial body weight, and any signs of distress including abnormalities in motility and respiration) was reached. At the endpoint, organs (such as the liver, kidney, gastrointestinal tract, pancreas, and spleen) and solid tumours dispersed throughout the abdominal region were collected from each mouse and processed for H&E staining as described previously [53]. Tissue sections were imaged using an Evos microscope FL Auto 2 cell imaging system (Thermo Fisher Scientific, Melbourne, Australia).

### 2.17. Statistical Analysis of the Data

The data obtained from all experiments were analysed statistically using the GraphPad Prism version 9.5.0. There was a minimum of 3 biological replicates per group/experiment. All the data are graphically demonstrated as mean ± standard deviation (SD). Significance differences were examined with respect to *p*-value < 0.05. For the comparison of data, either one-way ANOVA with multiple comparisons or Student’s *t*-test was used, which are stated with each of the dataset of graphs, respectively.

## 3. Results

### 3.1. Expression of Magmas in Ovarian Carcinomas and Benign Ovarian Tumours

**Figure 1:**
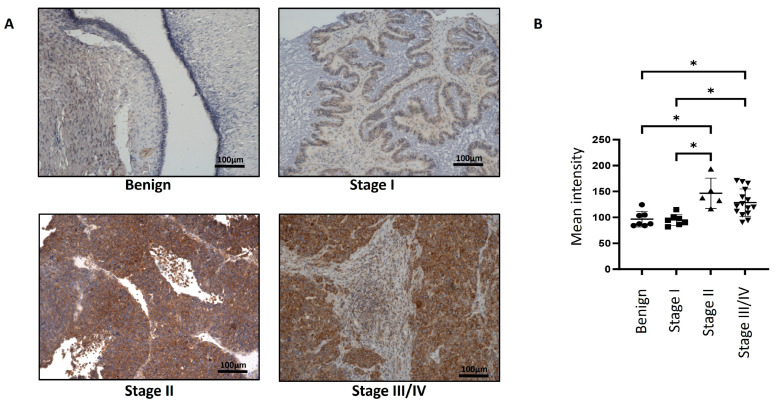
Expression of Magmas in primary ovarian tumours according to FIGO stages. (**A**) Representative images of Magmas staining in benign ovarian tumours (n = 7) and primary ovarian tumours (n = 26) at stages I, II, and III/IV. Magnification 20×; scale bar: 100 μm. (**B**) Graph represents the mean intensity of DAB positivity ± SD. Significance was determined by one-way ANOVA (Dunnett’s multiple comparison test) and indicated by * *p* < 0.05.

**Figure 2:**
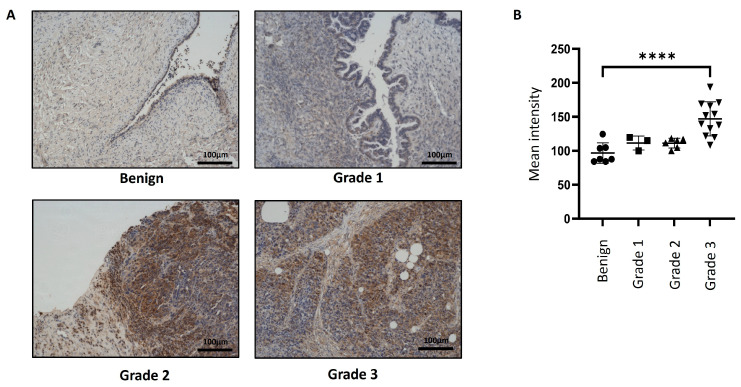
Expression of Magmas in primary ovarian tumours according to Silverberg grades. (**A**) Representative images of Magmas staining in benign ovarian tumours (n = 7) and primary ovarian tumours (n = 21) at grades 1, 2, and 3. The ungraded group was not included in this analysis. Magnification: 20×; scale bar: 100 μm. (**B**) Graph represents the mean intensity of DAB positivity. Values are mean ± SD with significance deduced using one-way ANOVA (Dunnett’s multiple comparison test) and indicated by **** *p* < 0.0001.

Immunohistochemistry staining of Magmas was performed on n = 9 serous benign and n = 28 serous ovarian tumour samples (Supplementary Table S1). In almost all tumours, Magmas expression was typically noted in the epithelial tumour cells, and a very low expression was noted in tumour stroma (Figure 1 and Figure 2). The differences in the expression of Magmas were analysed according to FIGO stages (Figure 1) and Silverberg grades (Figure 2) of ovarian tumours. In benign tumours, low expression of Magmas was noted on the surface epithelium of the ovaries. In a similar way, low expression of Magmas was noted on the surface epithelium of stage I and grade 1 ovarian tumours (Figure 1 and Figure 2). The expression of Magmas was significantly enhanced in stages II and III/IV ovarian tumours compared to benign and stage I tumours (Figure 1A,B). Consistent with that, both grade 3/4 tumours stained significantly high for Magmas expression compared to benign tumours (Figure 2A,B). Overall, the level of Magmas expression increased significantly in advanced stages and grades of ovarian tumours compared to benign and low-grade/stage tumours. Magmas expression was mainly confined to the epithelial cells of the tumours, and it was considerably enhanced in high grades and stages of tumours, while low grades and stages of tumours presented low Magmas expression.

### 3.2. The Expression of Magmas Is Inversely Related to ROS Levels in Ovarian Tumours

**Figure 3:**
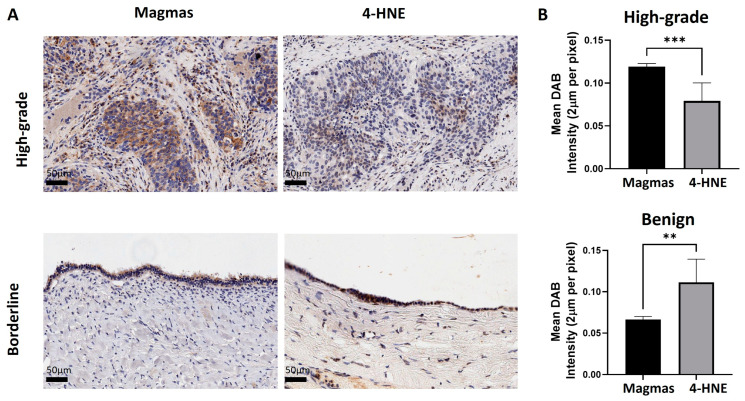
Expression of Magmas and 4-HNE in high-grade and benign ovarian tumours. (**A**) Representative images of Magmas and 4-HNE staining in high-grade (n = 3) and benign ovarian tumours (n = 3). Magnification: 40×, scale bar: 50 μm. (**B**) Graph represents mean DAB intensity (2 µm per pixel) ± SD. Significance was determined by a *t*-test (Mann–Whitney) and indicated by ** *p* < 0.01 and *** *p* < 0.001.

Magmas expression has been shown to eliminate ROS and promote a growth-friendly TME for tumour progression in response to oxidative and cytotoxic stress in yeast and human cancer models [35,37,38]. In this study, we compared the expression of Magmas with 4-Hydroxynonenal (4-HNE), a product produced by lipid peroxidation in cells in response to oxidative stress in high-grade (n = 3) and benign (n = 3) ovarian tumours, which had high and low expressions of Magmas (Figure 3). We show that high-grade tumours with high expressions of Magmas had low expressions of 4-HNE (Figure 3). Contrary to that, a benign tumour that had high expressions of 4-HNE showed very low expressions of Magmas, suggesting an inverse relationship between Magmas and 4-HNE expression in ovarian tumours (Figure 3). These observations are consistent with the previously identified ROS scavenging role of Magmas in yeast and human cancer models [35,37].

### 3.3. Magmas Was Expressed Variably in OC Cell Lines

**Figure 4:**
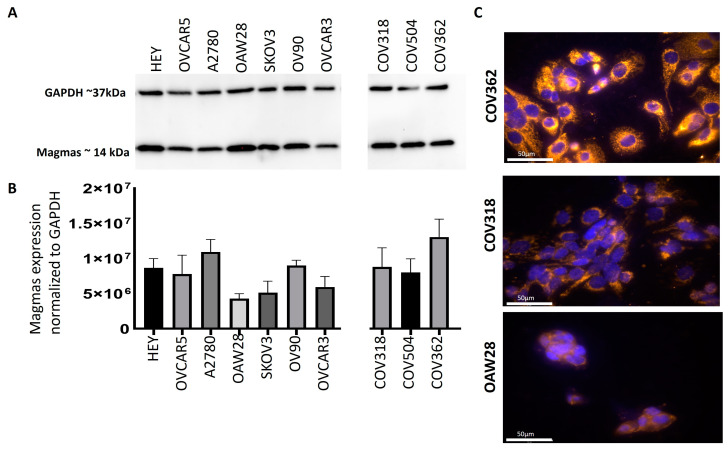
Expression of Magmas in OC cell lines. (**A**) Differential protein expression of Magmas in OC cell lines was deduced by WB as described in Methods. (**B**) The bar graphs represent the optical density of the Magmas band standardised to housekeeping protein GAPDH and are mean ± SD (n = 3) for each cell line collected at different split passages. (**C**) Protein expression of Magmas was evaluated in COV362, COV318, and OAW28 cell lines by IF using anti-Magmas rabbit monoclonal antibody as described in Methods. Staining was visualised using the secondary anti-rabbit Alexa 555 fluorescent-labelled antibody (orange) and nuclei were detected by DAPI (blue) staining. Magnification: 20×; scale bar: 50 µm.

The protein expression of Magmas was evaluated by WB in the cell lysates or the cells by IF on 10 different ovarian cancer cell lines originating from either solid tumours, ascites, or plural effusions of patients diagnosed with OC (Figure 4A–C, Supplementary Figures S1 and S2A). Of these cell lines, HEY, A2780, and ES2 (https://www.cellosaurus.org/, accessed 10 April 2024) were derived from solid tumours, while OVCAR5, OAW28, SKOV3, OV90, COV318, and OVCAR3 (https://www.cellosaurus.org/ accessed 10 April 2024) originated from ascites and COV504 and COV362 (https://www.cellosaurus.org/ accessed 10 April 2024) were developed from pleural effusion of OC patients (Table 1). Most of the OC cell lines tested had a consistent expression of Magmas, except SKOV3 and OAW28, which showed relatively a low expression of Magmas (Figure 4A,B). There was no difference in the expression pattern of Magmas in cell lines originating either from primary tumours, ascites, or pleural effusion.

The expression of Magmas was also evaluated in three OC cell lines by IF (Figure 4C). Cell lines with a high (COV362), medium (COV318), and low (OAW28) expression of Magmas obtained by WB were selected for the evaluation of Magmas expression by IF (Figure 4C). The level of expression of Magmas evaluated by IF was consistent with that observed by WB. The IF images demonstrated each cell line to have relatively good mitochondrial Magmas expression (yellow), located in the cytoplasm that was surrounded by the nuclei (blue) (Figure 4C). Although most of the Magmas expressions were confined to the mitochondria located in the cytoplasm, a small amount of Magmas expression, especially in the high-Magmas-expressing COV362 cell line, demonstrated vague nuclear expression, possibly being derived from mitochondrial DNA (Figure 4C). We also show expression patterns of Magmas in high-Magmas-expressing HEY, OVCAR5, and ES2 cell lines (Supplementary Figures S1 and S2A).

### 3.4. Determination of IC_50_ Values and the Effect of BT#9 on the Expression of Magmas in OC Cell Lines

**Figure 5:**
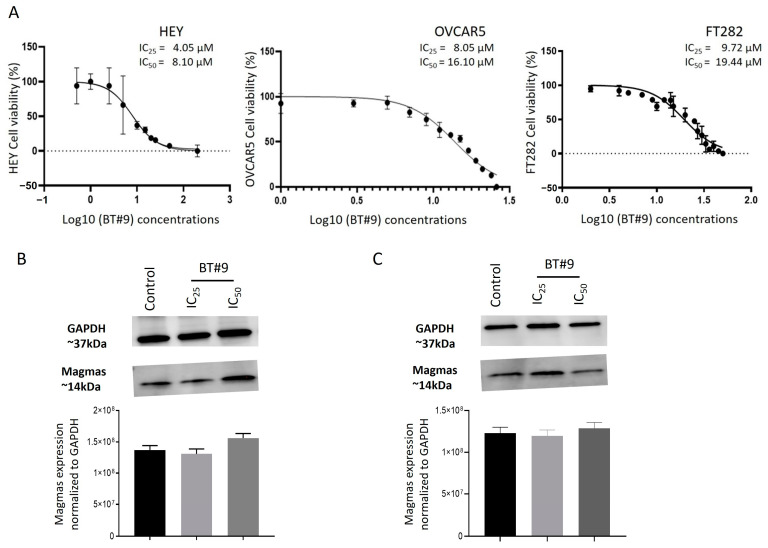
Effect of BT#9 on the IC_50_ values of HEY, OVCAR5, and FT282 cell lines. (**A**) Cell lines (HEY, OVCAR5, and FT282) were treated for 48 h with varying concentrations of BT#9, and their IC_50_ values (the concentration that kills 50% of the cells) were determined by the WST assay. Graphs are representative of three experiments performed in triplicate. (**B**) The protein expression of Magmas in HEY and (**C**) OVCAR5 cell lines treated with IC_25_ and IC_50_ concentrations of BT#9 for 24 h was evaluated by WB as described in Methods. Images represent three independent experiments performed in triplicate. The bar graphs represent the optical density of the Magmas band standardised to housekeeping protein GAPDH, and values are mean ± SD (n = 3) for each cell line collected at different split passages.

BT#9 is a small molecule inhibitor of Magmas, previously shown to bind to Magmas and inhibit its activity [43]. The IC_50_ values measure the 50% growth inhibitory concentration of cytotoxic drugs in cellular systems. The IC_50_ value of BT#9 in mesenchymal-fibroblast-like HEY and ES2 cell lines derived from primary tumours were significantly lower (nearly 40% less) than those observed in epithelial–mesenchymal mixed OVCAR5 cell lines derived from ascites (Figure 5A and Supplementary Figure S2B). Interestingly, the IC_50_ value of BT#9 in FT282 cells derived from normal epithelial fallopian tubes was significantly higher than that of HEY, ES2, and OVCAR5 OC cell lines (Figure 5A).

To determine if the growth inhibitory effect of BT#9 on OC cells has any effect on the expression of Magmas, WB was performed on HEY and OVCAR5 cells treated with BT#9 for 24 h at the concentrations of IC_25_ (25% growth inhibition) and IC_50_ (50% growth inhibition) (Figure 5B,C). There was no change in the expression of Magmas on either cell line treated with BT#9 at the concentrations indicated above (Figure 5B,C). This indicates that the effect of BT#9 on OC cells is independent of a change in Magmas expression.

### 3.5. BT#9 Induces Both Apoptosis and Necrosis in OC Cell Lines

**Figure 6:**
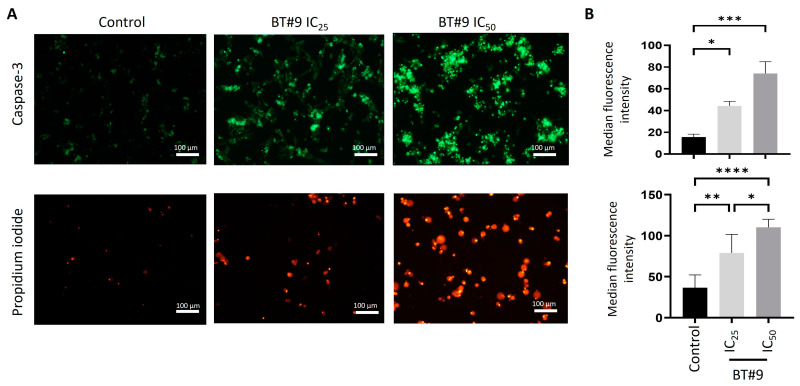
Effect of BT#9 on the apoptotic and necrotic response of the HEY cell line. (**A**) Immunofluorescence images of active caspase 3/7 and PI-stained HEY cells were captured after treatment with IC_25_ and IC_50_ values of BT#9 for 12 h as described in Methods. Images represent three independent experiments performed in triplicate. Magnification: 20×; scale bar: 100 μm. (**B**) Bar graphs represent the median fluorescence intensity of caspase 3/7 and PI staining at the respective BT#9 concentrations for n = 3 experiments performed in triplicate. Values are mean ± SD with significance deduced using one-way ANOVA (Tukey’s multiple comparison test) and indicated by **** *p* < 0.0001, *** *p* < 0.001, ** *p* < 0.01, and * *p* < 0.05.

Most cytotoxic compounds kill cancer cells either by apoptosis or necrosis, or they may apply both methods. To determine if BT#9 follows the apoptotic or necrotic form of cell killing, we performed active caspase 3/7 [56] and PI [57] staining of OC HEY and OVCAR5 cell lines after 12 h treatment with IC_25_ and IC_50_ values of BT#9. We show that BT#9 can induce both apoptotic and necrotic forms of cell death in both HEY (Figure 6) and OVCAR5 (Supplementary Figure S3) cells, as indicated by active caspase 3/7 and PI staining. In control cells, very few dead cells were observed, which can be due to cell processing during culturing (Figure 6 and Supplementary Figure S3). However, the staining of apoptotic and necrotic dead cells by active caspase 3/7 and PI staining increased significantly after the cells were treated with IC_25_ or IC_50_ concentrations of BT#9 for 12 h (Figure 6 and Supplementary Figure S3). The number of dead cells increased in IC_50_ compared to IC_25_ BT#9 treatment for the same length of time. This indicates that BT#9 induced both apoptotic and necrotic forms of cell death in OC cells in a dose-dependent manner. We also show 12 h brightfield images of the HEY cell line treated with IC_25_ and IC_50_ BT#9 concentrations stained with caspase 3/7 and PI (Supplementary Figure S4). The images are consistent with the IF images, which show a progressive increase in active caspase 3/7 and PI-stained cells treated with BT#9 IC_50_ concentrations for 12 h compared to cells treated with IC_25_ concentrations.

### 3.6. BT#9 Induces Loss of Mitochondrial Membrane Potential in OC Cell Lines: TMRM Assay

**Figure 7:**
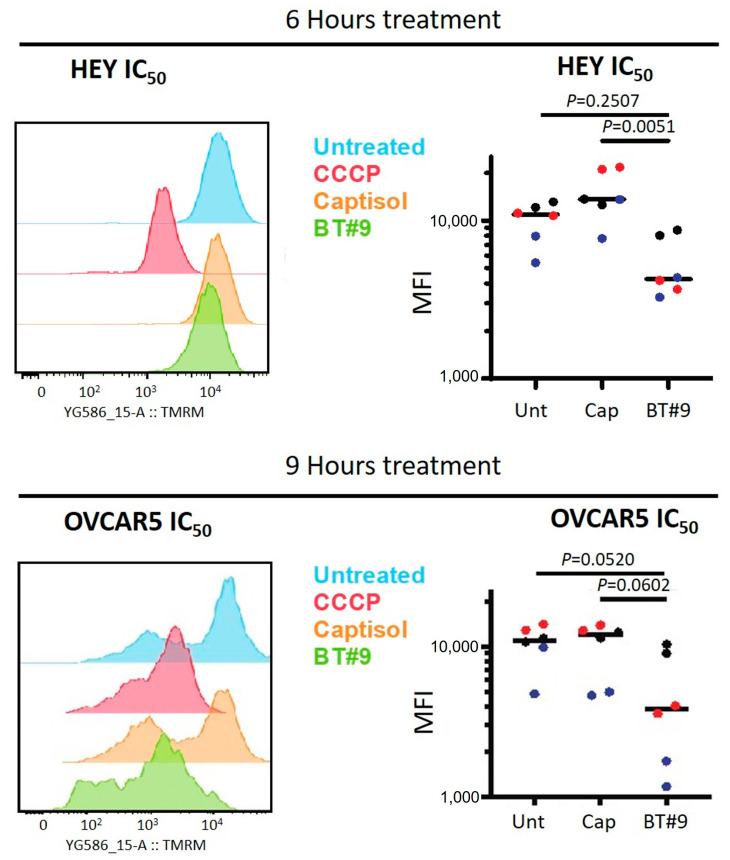
Effect of BT#9 treatment on mitochondrial membrane potential in HEY cells. Representative histograms showing fluorescence intensity of tetramethylrhodamine methyl ester (TMRM) from one experiment of HEY cell line treatment with BT#9 IC_50_ at 6 h and OVCAR5 cell line treated with IC_50_ BT#9 for 9 h. Each treatment included an untreated control, a captisol-treated control, and a control with carbonyl cyanide 3-chlorophenylhydrazone (CCCP) added to block the mitochondrial uptake of TMRM. Data represent the MFI of three passages of each cell line performed in duplicate cultures, with dot colours representing the same passage. Significance was determined using a Kruskal–Wallis multiple comparisons test.

To determine if BT#9 induces loss of mitochondrial membrane potential, a TMRM assay was performed in both HEY and OVCAR5 cell lines in response to BT#9 treatment (Figure 7). There was a downward shift in the mitochondrial potential with IC_50_ concentration of BT#9 treatment at 6 h for the HEY cell line and 9 h for the OVCAR5 cell line (Figure 7).

### 3.7. BT#9 Induced the Production of ROS Levels in OC Cell Lines: DCFDA Assay

**Figure 8:**
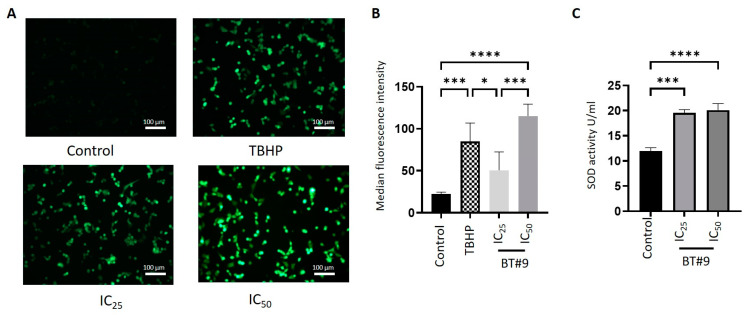
Effect of BT#9 on ROS production in HEY cells. (**A**) The HEY cell line was treated with IC_25_ and IC_50_ concentrations of BT#9, and intracellular ROS production was measured according to the manufacturer’s instructions, as indicated in the DCFDA ROS detection assay kit. ROS generation was observed under a fluorescence microscope at 20× magnification, and the scale bar was 100 µm. (**B**) Bar graphs represent the median fluorescence intensity of DCFDA. Values are mean ± SD with significance deduced using one-way ANOVA (Tukey’s multiple comparisons test) and indicated by **** *p* < 0.0001, *** *p* < 0.001, * *p* < 0.05 when compared to untreated cells. Tert-butyl hydrogen peroxide (TBHP, 150 µM) acts as a positive control and mimics ROS activity to oxidise DCFDA to fluorescent DCF. (**C**) The mitochondrial SOD level in the HEY cell line treated with IC_25_ and IC_50_ BT#9 concentrations was determined by the SOD assay kit, as described in Methods. The experiment was repeated three times, and data are presented as n = 3. Values are mean ± SD with significance deduced using one-way ANOVA (Dunnett’s multiple comparisons test) and indicated by **** *p* ≤ 0.0001 and *** *p* ≤ 0.001, when compared to untreated cells.

The total cellular ROS levels in HEY and OVCAR5 OC cell lines in response to IC_25_ and IC_50_ BT#9 treatments were measured by IF using a DCFDA stain (Figure 8 and Supplementary Figure S5). In both cell lines, a consistent BT#9 concentration-dependent increase in ROS level was observed by DCFDA staining measured by IF (Figure 8A,B and Supplementary Figure S5A,B). Even though there was an increase in ROS production with IC_25_ BT#9 treatment at 12 h, significance was only observed at IC_50_ BT#9 treatment (Figure 8A and Supplementary Figure S5A).

To measure if the mitochondria are the major source of ROS levels, superoxide production was measured by the SOD assay, which measures the enzymic activity of superoxide dismutase required to convert superoxide anion (O_2_^−^) to water (Figure 8C and Supplementary Figure S5C). The level of SOD activity was enhanced by BT#9 treatment in both cell lines, indicating an enhanced level of O_2_^−^ production in response to BT#9 treatment and quenching of that effect by SOD enzyme activity.

### 3.8. BT#9-Treated OC Cells Showed Lower Bioenergetics Compared to Their Untreated Control Cells

**Figure 9:**
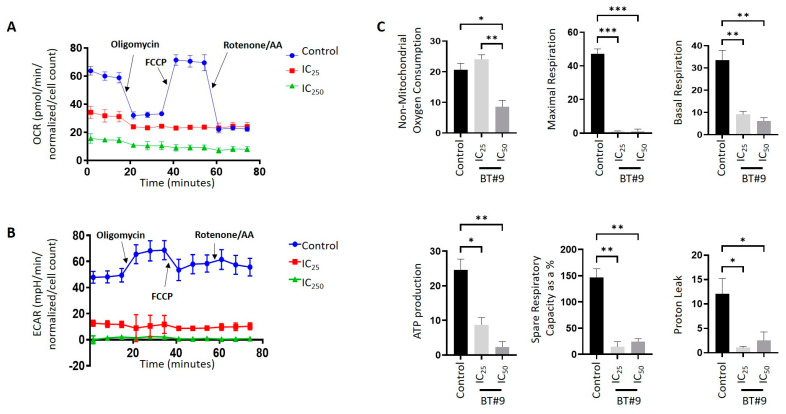
Effect of BT#9 on the cellular bioenergetics of the OVCAR5 cell line. Cellular bioenergetics was evaluated by a Mito stress assay in response to treatments with IC_25_ and IC_50_ values of BT#9 for 12 h. Representative graph of measurements of (**A**) the oxygen consumption rate (OCR) and (**B**) the extracellular acidification rate (ECAR) following the sequential addition of oligomycin (ETC complex V inhibitor, inhibits ATP synthase), FCCP (inhibitor of mitochondrial membrane potential), and Rotenone (ETC complex 1 inhibitor) as described in Methods. (**C**) Bar graphs represent the non-mitochondrial oxygen consumption rate, maximal respiration, basal respiration, ATP production, spare respiratory capacity, and proton leak, measured using the parameters described in (**A**). Each parameter was normalised to total protein concentration. The experiment was repeated three times, and data are presented as n = 3; values are mean ± SD, with significance deduced using one-way ANOVA (Tukey’s multiple comparison test) and indicated by *** *p* < 0.001, ** *p* < 0.01, and * *p* < 0.05 when compared to OVCAR5 untreated cells.

Cancer cells regularly undergo altered metabolism in response to adverse conditions in the TME [58]. To understand how OC cells change their energy pattern in response to BT#9 treatment, we investigated the cellular bioenergetic pattern of control and 12 h IC_25_ and IC_50_ BT#9-treated OVCAR5 and HEY OC cell lines using a Mito stress assay, which measures the oxygen consumption rate (OCR), mitochondrial oxidative phosphorylation (OXPHOS), and extracellular acidification rate (ECAR), a measure of glycolysis in the presence of ETC inhibitors, oligomycin (ETC complex V inhibitor, inhibits ATP synthase), FCCP (inhibitor of mitochondrial membrane potential), Antimycin A (inhibitor of complex III), and Rotenone (ETC complex 1 inhibitor). The control OVCAR5 cell line showed steady OCR and ECAR baseline levels, which were lower for both OCR and ECAR in OVCAR5 BT#9-treated cells (Figure 9A,B). Upon oligomycin injection, in control OVCAR5 cells, the OCR level drastically decreased, consistent with an increase in ECAR levels, indicating a shift towards glycolytic flux (Figure 9B). On the injection of FCCP, an increase in OCR response was noted in control cells (Figure 9A). However, no such response was noted in BT#9-treated cells (Figure 9A). The injection of Rotenone drastically reduced the OCR response in control cells, with no such change in response noted in BT#9-treated cells. The enhanced ECAR response after injection of oligomycin was retained in control cells, which were diminished after treatment with FCCP and Rotenone, suggesting the reliance of these cells on glycolytic flux in the absence of OXPHOS (Figure 9B). However, no changes in ECAR response were noted in BT#9-treated cells (Figure 9B).

Consistent with the above findings, the basal and maximal respiration, ATP production, spare respiratory capacity, and proton leak of the OVCAR5 cell line were significantly decreased with BT#9 treatments (Figure 9C). Simultaneously, a decrease in non-mitochondrial oxygen consumption was observed in OVCAR5 cells treated with IC_50_ BT#9, while it was maintained in IC_25_ BT#9-treated OVCAR5 cells (Figure 9C).

The control HEY cell line responded similarly to the OVCAR5 cell line with respect to OCR and ECAR responses upon treatment with the Mito stress inhibitors (oligomycin, FCCP, and Rotenone/Antimycin A) (Supplementary Figure S6A,B). However, in the BT#9-treated HEY cells, there was a limited change at both IC_25_ and IC_50_ treatments (Supplementary Figure S6A,B). Following BT#9 treatments, the basal and maximal respiration, ATP production, and non-mitochondrial oxygen consumption were affected by both IC_25_ and IC_50_ BT#9 treatments. However, the spare respiratory capacity and proton leak were compromised only with the IC_50_ BT#9 treatment, showing no impact with the IC_25_ BT#9 treatment (Supplementary Figure S6C).

### 3.9. BT#9 Inhibits the Proliferation and Migration of OC Cell Lines

**Figure 10:**
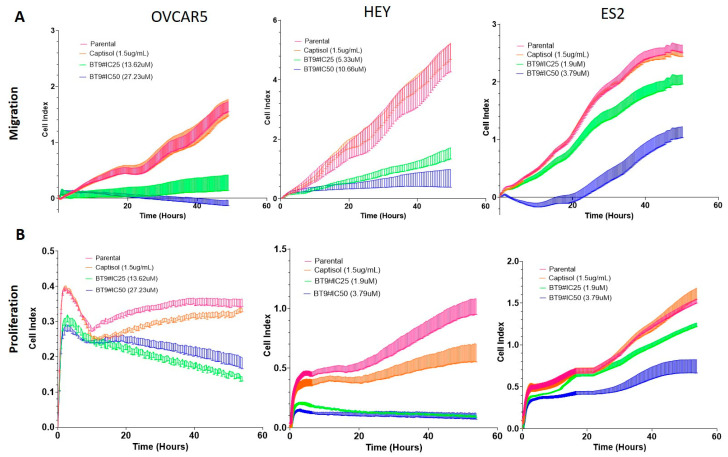
The proliferative and migratory abilities of HEY, OVCAR5, and ES2 cell lines in response to BT#9 treatments. HEY, OVCAR5, and ES2 cell lines were treated with IC_25_ and IC_50_ concentrations of BT#9, and their (**A**) proliferative and (**B**) migratory responses were evaluated by the xCELLigence RTCA DP (dual-purpose) instrument, as described in Methods. The experiment was repeated two times, and the real-time data from one representative experiment performed in duplicate are presented.

The proliferation and migration properties of OVCAR5, HEY, and ES2 cell lines were assessed by real-time cell analyses (RTCAs) using Agilent xCELLigence equipment. For the proliferation assay in all three cell lines, there was a gradual increase in proliferation with time (Figure 10A). The proliferation rate in control untreated and captisol groups in both HEY and OVCAR5 cell lines progressively increased within 50 h, but in the ES2 cell line, it plateaued within that time frame (Figure 10A). Both IC_25_ and IC_50_ BT#9 treatments of HEY, OVCAR5, and ES2 cells followed a similar pattern where an inhibited proliferation prototype was observed compared to control untreated and captisol-treated cells (Figure 10A). This inhibition of proliferation by BT#9 treatment was observed in the early hrs, which persisted over the 50 h time frame.

For the migration assay, a gradual increase in migration was observed over the period of 40–50 h in control and captisol-treated OVCAR5, HEY, and ES2 cell lines (Figure 10B). There was an inhibition of that migratory response with BT#9 treatments at both IC_50_ and IC_25_ concentrations and BT#9 treatment (Figure 10B). Interestingly, in all three cell lines, there was an initial increase in migration within the first few hrs, irrespective of treatment with BT#9. This may be due to the chemotaxis effect of serum in the lower migration chambers.

### 3.10. Chemosensitivity of the OC Cell Lines to Paclitaxel in Response to BT#9 Treatment

**Figure 11:**
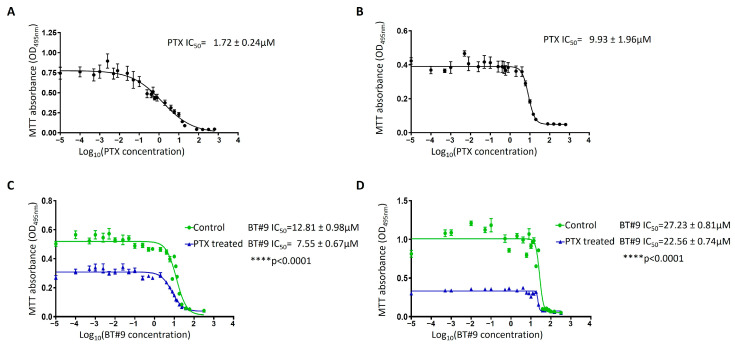
Combined effect of BT#9 and PTX on HEY and OVCAR5 cell lines. (**A**) HEY and (**B**) OVCAR5 cell lines were treated for 48 h with varying concentrations of PTX to determine the IC_50_ value by the MTT assay, as described in Methods. (**C**) HEY and (**D**) OVCAR5 cells were treated with varying Log10 concentrations of BT#9 (green line) or with the PTX IC_50_ amount (obtained in **A** and **B**) plus varying Log10 concentrations of BT#9 (blue line). Cell viability was obtained by the MTT assay. Data are representative of three independent experiments using cells at different passages treated in triplicate. Values are mean ± SD, with significance deduced using Student’s *t*-test, indicated by **** *p* < 0.0001, when compared between cells treated with BT#9 and PTX (IC_50_) + BT#9.

In this study, we first treated the HEY and OVCAR5 cells at different concentrations of PTX to obtain the appropriate IC_50_ value for each cell line (Figure 11A,B). The IC_50_ values of PTX for each cell line were then used to determine the IC_50_ value of the combination of PTX+BT#9 treatment. Following this, we demonstrate that both HEY and OVCAR5 OC cell lines have significantly enhanced sensitivities to BT#9 concentrations in combination with PTX (Figure 11C,D). In the HEY cell line previously treated with the IC_50_ PTX and then treated with an increasing Log10 concentration of BT#9, the initial BT#9 IC_50_ value of 12.81 was decreased to a PTX+BT#9 IC_50_ value of 7.55 (*p* < 0.0001; Figure 11C). Consistent with that, the OVCAR5 cell line had a BT#9 IC_50_ of 27.23, which was reduced to an IC_50_ of 22.56 in the presence of an increasing Log10 concentration of BT#9 plus IC_50_ PTX (*p* < 0.0001; Figure 11D). These observations suggest that the addition of BT#9 can enhance the sensitivity of OC cell lines to PTX treatment.

The differences in the IC_50_ values of BT#9 in HEY and OVCAR5 cell lines in this experiment compared to the IC_50_ values in Figure 5 may be due to differences in the passages of the cell lines used, and the batches of BT#9 used as these experiments were performed months apart. In addition, different experimental outlay and methods were used; Figure 5 used the WST-1 assay for 24 h treatment with BT#9, while in Figure 11, the MTT assay was performed for 48 h BT#9-treated cells.

### 3.11. The Effect of BT#9 on HEY Cell-Induced Tumours in Mice: Tumour Burden and Survival Studies

We investigated the in vivo antitumourigenic effect of BT#9 by examining the tumour burden as well as the survival of mice upon daily oral gavage of BT#9 in response to tumour imitation by injecting HEY cells intraperitoneally. In our previous studies, we have shown that the HEY cell line produces a robust tumour burden intraperitoneally in nude mice [55]. In the first part of this study, we investigated the tumour burden in control, captisol (vehicle control), and BT#9-treated (daily oral gavage at 30 mg/kg body weight) mice. All mice were culled at the endpoint of the untreated mice, and their tumour burden was measured. For the survival study, BT#9 oral gavages in one group of mice continued until the experimental endpoint.

**Figure 12:**
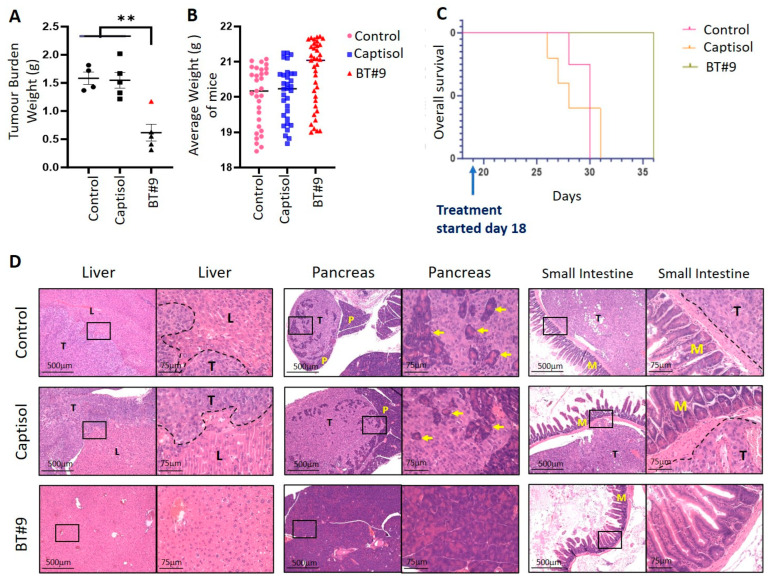
Tumour burden, mice average weight, and Kaplan–Meier survival curves of mice injected with the HEY cell line. (**A**) The control (n = 5), captisol-treated (n = 5), and BT#9-treated (daily oral gavage, 30 mg/kg body weight) mice groups were injected IP with the HEY cell line. Treatment with daily oral gavages of BT#9 (30 mg/kg body weight) was initiated 18 days post-inoculation with HEY cells. At the endpoint of control untreated mice at 30 days post-inoculation, all mice were subjected to euthanasia and were dissected, and their tumours were excised and weighed. The graph demonstrates the average tumour burden obtained from the mice in each group. Values are mean ± SD, and significance was deduced using one-way ANOVA (Tukey’s multiple comparison test) and indicated by ** *p* < 0.01 compared to control untreated mice. The red dot in the BT#9 group indicates the mouse that had a higher tumour burden that metastasised to peritoneal organs. (**B**) Average body weight of mice (g) in each group, each day, through the progression of the experiment. (**C**) Kaplan–Meier survival curves of mice injected with the HEY cell line and treated as indicated in (**A**). (**D**) H&E staining of organs from mice injected IP with HEY cells and treated as described in Figure 12A. The black dotted line demarcates the invading tumour (T) from the non-cancerous liver (L), pancreas (P), or small intestine mucosa (M) within each organ. Yellow arrows within the pancreas photomicrographs indicate normal pancreatic acinar surrounded by tumour. Photomicrographs are ×10 magnification (scale bar = 500 μm) and ×40 magnification (scale bar = 75 μm); the black rectangle within the ×10 image indicates ×40 image capture. In Supplementary Figure S7, we present images of live mice taken on day 28 for all three groups: control, captisol, and BT#9 treated. The images indicate the extent of ovarian cancer tumour burden based on the extensive abdominal distention of the control and captisol group of mice compared to no abdominal distention in the BT#9-treated mice group. In addition, BT#9-treated mice showed an extra flap of skin, indicative of a lack of distention of the abdomen compared to control and captisol groups, where no visible flap of skin was observed.

In brief, both for tumour burden and survival studies, 5 × 10^6^ HEY cells were injected peritoneally into 20 nude mice. Mice were divided into four groups: control (n = 5), captisol (n = 5) group 1, BT#9 treatment (n = 5), and group, 2 BT#9 treatment (n = 5). Eighteen days post-injection, when the intraperitoneal tumour could be detected with palpation, daily oral gavage of BT#9 treatment (30 mg/kg body weight) was started in the BT#9 treatment groups, while the captisol group received an equal volume of captisol (100 μL) as the BT#9 group. Thirty to thirty-one days post-inoculation of HEY cells, treated mice [n = 5, captisol treated, and group 1, n = 5 BT#9 treated] and control untreated mice were subjected to euthanasia at the humane endpoint of the control untreated mice [53]. Treatments with captisol and BT#9 were endured well by mice without any adverse toxicological signs throughout the experiment. Control untreated mice and mice treated with captisol showed a significantly much bigger tumour burden than the mice group receiving oral gavages of BT#9 (Figure 12A). Four mice in the group receiving oral gavages of BT#9 produced a significantly smaller tumour burden, while the fifth one (indicated in red in Figure 12A) had a much higher tumour burden. Overall, fewer, smaller tumours were found in each mouse in the BT#9 treatment group compared to control untreated and captisol-treated mice. The tumour burden in each group reflects the total weight of all visible tumours collected from the peritoneal cavity of each mouse in different groups (Figure 12A). The tumour burden in the BT#9 group includes all tumour weight from all the mice, including the outlier mice (indicated in red in Figure 12A) in that group.

All mice in each group were active until the endpoint of the experiment. The average body weight of mice in each group is presented in Figure 12B. There was no significant change in the body weight of control and captisol-treated mice, which survived for 30–31 days, compared to BT#9-treated groups, which survived for 36 days (Figure 12B). An insignificant increase in the BT#9-treated group may be due to their longer survival time of 36 days compared to 30–31 days in untreated control and captisol-treated mice. In addition, 4/5 mice in the BT#9-treated group were free of metastasis, which may also contribute to the body weight through eating normally.

Daily oral gavage of BT#9 was continued in the remaining n = 5 mice that were previously being treated with BT#9. These mice survived longer than the control and captisol-treated groups. Kaplan–Meier analysis of survival revealed that control untreated and captisol control mice groups survived 30–31 days post-inoculation of HEY cells, while the fourth group of BT#9-treated mice was alive 36 days post-inoculation of HEY cells (Figure 12C). The experiment at this stage had to be discontinued due to COVID-19 restrictions. Overall, group 4 mice that received ongoing daily BT#9 treatment survived much longer and could have survived more if the experiment had been continued (Figure 12C).

To determine whether the administration of BT#9 had any in vivo effect on tumour metastasis, mice organs (liver, pancreas, spleen, kidney, small bowel, and large bowel) were collected and subjected to H&E staining (Figure 12D). Upon dissection, multiple macroscopic and microscopic tumour deposits were observed in the abdomen, with some deposits penetrating primarily the liver, pancreas, and bowel, and several smaller tumour nodules were also found throughout the peritoneal cavity of the control untreated and captisol-treated mice. However, in the BT#9 treatment group, small tumours were localised mainly in the abdominal cavity, and no penetration of tumour was observed in any of the organs in n = 4 mice (Figure 12D). Only one mouse that showed a comparatively higher tumour burden in the BT#9 group (in red) (Figure 12A) had tumours penetrating the liver and pancreas, as observed in control mice. The collected tumours were weighed and preserved in formalin or Trizol or snap-frozen for mRNA and proteomics studies.

In Supplementary Figure S8, we show that BT#9 treatment had no effect on the mRNA expression of Magmas compared to controls (PBS and captisol treatments). This result is consistent with no change in Magmas protein expression in HEY and OVCAR5 cell lines treated with IC_25_ and IC_50_ concentrations of BT#9 treatments for 48 h (Figure 5B). 

**Figure 13:**
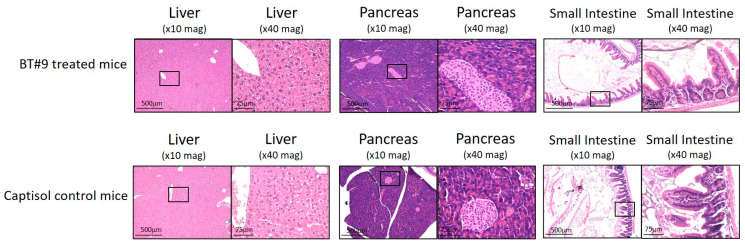
H&E staining of organs from mice given oral gavages of BT#9 without tumour induction. Photomicrographs of the liver, pancreas, and small intestine from BT#9-treated mice (n = 3) and untreated mice (n = 2). Photomicrographs are ×10 magnification (scale bar = 500 μm) and ×40 magnification (scale bar = 75 μm); the black rectangle within the ×10 image indicates ×40 image capture.

To determine if BT#9 by itself had any toxic effect on mouse organs without tumour induction, two groups of mice, the control group (n = 2) and the BT#9-treated group (n = 3), were organised. In the BT#9-treated group, daily oral gavage treatment was continued for 23 days, after which both the control and BT#9 treatment groups were euthanised, organs were collected, and H&E was performed. No toxicity of BT#9 was observed in any of the mouse organs by H&E staining (Figure 13).

## 4. Discussion

Mitochondria are the ‘energy centre’ from which cells derive 90% of their fuel in the form of ATP [58,59]. Mitochondria also facilitates free radical scavenging, intercellular calcium regulation, and the release of protein that activates the caspase family of proteases for the induction of apoptosis, and it changes in the membrane potential to facilitate the transfer of ions through various channels and transporters (uniporters, cotransporters, and pumps) [60]. Mitochondrial dynamics is modulated by proteins, which makes the inner and outer membranes of mitochondria and an intermembrane space, and it relies on the regulatory signals they receive in response to the changing cues in the cellular environment [59]. The inner membrane of mitochondria where Magmas is located consists of the mitochondrial matrix and the cristae, which contain the enzymes for mitochondrial respiration and ATP synthesis [61]. As such, the mitochondrial inner membrane is essential to maintain cellular bioenergetics, which is critical to sustaining a balance between cell growth and death [62].

The recent literature suggests that the number of mitochondria is enhanced with OC progression, the mitochondrial cristae morphology is altered, and there are changes in mitochondrial DNA (mtDNA) content and mtDNA mutations compared to normal ovarian tissues [20,21,63]. These observations implicate aberrant mitochondrial functionality associated with tumourigenesis in OC and suggest that targeting mitochondria can potentially be an effective therapeutic strategy for OC treatment. The current mitochondria-targeting anticancer drugs have shown limited effectiveness in preclinical studies of cancer [64]. Hence, it is necessary to explore novel mitochondria-targeting agents that may provide optimal effective therapy in treating OC patients who currently have very limited options.

In the current study, we extensively analysed the expression of the inner mitochondrial membrane protein, Magmas, in serous ovarian tumours and OC cell lines. We report that the expression of Magmas is enhanced significantly in high FIGO stages and Silverberg grades of OC tumours compared to benign and low-stage/-grade tumours. The fact that a high expression of Magmas coincides with a low expression of 4-HNE in high-grade tumours and that a high expression of 4-HNE is consistent with a low expression of Magmas in benign OC tumours, indicates that Magmas expression essentially is critical to sustaining the redox balance in ovarian tumours, as reported previously in the yeast model [35]. As increased production of mitochondrion-originated ROS is essential to drive malignant progression, it should be recognised that preserving the redox balance is essential for cancer cell proliferation, sustenance, and dissemination. Due to extensive tumour bulk, increased congestion pressure of ascites, and an upsurge of hypoxia, the progressive high-grade/-stage ovarian tumours are under increasing oxidative stress. In that scenario, a high expression of Magmas in high grades/stages of ovarian tumours may provide a protective shield from ROS-mediated harmful effects, thus supporting and promoting a sustainable TME for cancer progression. These observations are consistent with what was observed in the yeast model, where siRNA knockdown of Magmas caused a significant increase in ROS production, while the overexpression of Magmas rescued the dying cells from oxidative stress [35]. These observations were also replicated in a pituitary adenoma cell line model treated with an apoptosis-inducing PKC inhibitor and staurosporine, where the overexpression of Magmas rescued the cells from the apoptotic effect of staurosporine [36]. Consistent with these findings, we also report a significantly high expression of Magmas in OC cell lines, irrespective of their origin from primary tumours, ascites, or pleural effusions. There was a marginal deviation in the level of detectable Magmas protein expression in the eleven cell lines studied, suggesting that Magmas is intrinsically expressed in OC cell lines.

We next explored the effect of a small molecule Magmas inhibitor, BT#9, in vitro in two ovarian cancer cell lines, HEY and OVCAR5, and a cell line derived from a normal fallopian tube, FT282. BT#9 reduced the viability of all three cell lines in a dose-dependent manner. However, even though the expression of Magmas was the same in both HEY and OVCAR5 OC cell lines, the IC_50_ values for BT#9 were significantly different, with OVCAR5 cells demonstrating more resistance to BT#9 treatment compared to HEY cells. This can be due to differences in the intracellular level of ROS in these cells driven by phenotypic differences and needs further investigation. As HEY cells are inherently mesenchymal in morphology while OVCAR5 is a mixed population of epithelial and mesenchymal cells, the intracellular level of ROS in the HEY cell line is likely to be different than the OVCAR5 cell line. As BT#9 kills cells by binding to Magmas and disrupting the TIM23 complex, this may result in the loss of protein transfer in the inner mitochondrial membrane, resulting in perturbed mitochondrial respiration and decreased ATP synthesis. However, the Magmas–TIM23 complex has also been shown to be part of electron transport supercomplexes, complex IV, the perturbation of which may also affect the electron transport chain, resulting in enhanced intracellular ROS levels [31,65]. The paradoxical role of the intracellular ROS level may hold a delicate balance between BT#9-treatment-induced cancer cell death and survival. Interestingly, in the same experiment, BT#9 showed much less effectiveness in normal fallopian tube-derived FT282 cells compared to both HEY and OVCAR5 cell lines, which is consistent with the observations in osteosacrcoma, leukemia, lymphoma, prostate cancer, and brain tumour cell lines, where the tumour cell lines responded more effectively to BT#9 compared to the normal cell lines of the same origin [44]. In that context, in normal cells, proliferative signals keep a balanced redox range within the cells that allows reversible protein oxidation to occur. However, in cancer, due to aberrant signalling caused by enhanced ROS production, the redox range is shifted towards highly oxidising states, which can lead to oxidative stress and cell death. This process can be accelerated by ROS-producing agents, like BT#9, leading to cumulative cell death in cancer cells.

Protein expression analysis by Western blot showed that the cytotoxicity of BT#9 towards OC cells was independent of Magmas expression, as no loss of Magmas protein expression in BT#9-treated HEY and OVCAR5 cell lines at IC_25_ and IC_50_ concentrations after 24 h treatment could be detected. These results contradict what has recently been shown in prostate cancer cell line DU145, where BT#9 at a concentration of 10 μM after 24 h of treatment downregulated the expression of Magmas [37]. The inconsistency in these results may stem from the differences in the cell lines used or the methodology used for WB analysis. In our study, cell lysates of BT#9-treated residual viable cells were used to validate Magmas expression, while in the other study [37], total cell lysates may have been used.

Due to the mitochondrial localisation of Magmas and the dominant role of mitochondria in apoptosis, we next evaluated if BT#9-induced cellular death in OC cells predominantly used the apoptotic pathway. As active caspases 3/7 are the key proteases that are activated during the early stages of apoptosis, we stained BT#9-treated HEY and OVCAR5 cells for active caspase 3/7 staining and evaluated the results by IF methods. Active caspase 3/7 staining of 12 h BT#9-treated OC cells predominantly showed that BT#9 induced apoptotic cell death in both HEY and OVCAR5 cells in a dose-dependent manner, as more staining was observed at IC_50_ BT#9 concentration compared to IC_25_ concentration. However, BT#9 also induced an unprogrammed form of necrotic cell death, as evidenced by PI staining of both HEY and OVCAR5 cells after 12 h of BT#9 treatment. In summary, BT#9-induced cancer cell toxicity involves both apoptosis and necrotic pathways. These observations are consistent with the results seen in prostate cancer cells [37].

To further explore if the effect of BT#9 on cancer cell death is associated with a loss of mitochondrial membrane potential, a TMRM assay was carried out. In both HEY and OVCAR5 cell lines, the mitochondrial membrane potential was compromised by IC_50_ BT#9 concentration within 6–9 h. The loss of mitochondrial potential in HEY cells within 6 h compared to a similar phenomenon occurring in OVCAR5 cells within 9 h can be due to differences in the intracellular level of ROS in these cells driven by phenotypic differences, as explained before, and this needs further investigation. Consistent with that, we demonstrate that BT#9 significantly enhanced the intracellular levels of ROS in both HEY and OVCAR5 cell lines within the first 12 h. There was, however, a slight insignificant increase in the level of ROS production with IC_25_ BT#9 concentration in both cell lines. This was consistent with the significantly enhanced activity of ROS scavenging enzyme SOD activity in both HEY and OVCAR5 cell lines. This may occur as a compensatory mechanism to protect the BT#9-treated residual viable cells from the harmful effect of ROS.

Our study also shows that Magmas inhibition by BT#9 at both IC_25_ and IC_50_ concentrations resulted in impaired respiratory function within the first 12 h, as indicated by reduced OCR and ECAR in both HEY and OVCAR5 cell lines compared to control cells. In addition, the non-mitochondrial oxygen consumption was only compromised by BT#9 treatment in the HEY cell line, but the spare respiratory capacity was inhibited by BT#9 treatment in both cell lines. These changes in oxygen availability can have drastic effects on the viability and functions of cells, consistent with reduced proliferation and migration, as evidenced by the treatment of both IC_25_ and IC_50_ concentrations of BT#9 in HEY, OVCAR5, and ES2 cell lines. These results are consistent with previous studies on the effect of BT#9 on the proliferation and migration of glioma cells [38]. It is interesting to note that the inhibitory effect of BT#9 at both IC_25_ and IC_50_ concentrations on cellular proliferation was rapid and happened within the first few hrs (10–12 h) and coincided with the caspase 3/7 and PI staining of cells and the ROS production and impaired respiration in cells. However, a lack of mitochondrial membrane permeability by BT#9 at IC_25_ concentrations within the first 6–9 h may suggest that BT#9 initially affects the respiratory chain of cells, resulting in increased ROS production and leading to concomitant cell death. Our data indicate that the effect on mitochondrial membrane permeability may occur secondary to initial cell death induced by increased ROS production, which simultaneously adds to the cellular death process. In short, BT#9’s primary initial target is the deconvolution of the cellular respiratory chain, resulting in increased ROS production, which results in early cell death. This is later followed by its effect on mitochondrial membrane permeability, which adds to the apoptotic/necrotic processes, as shown by caspase 3/7 and PI staining. These observations are consistent with previous reports on the Drosophila system, which showed that treatment of Drosophila Schneider 2 (S2) cells with a small-molecule Magmas inhibitor (SMMI or BT#9) reduced cell proliferation and increased ROS levels within 1 h, while the loss of mitochondrial permeability was observed at a later stage of 2 h and onwards [31]. These observations, however, require further evaluation in future studies.

Furthermore, we show that the BT#9 treatment of both HEY and OVCAR5 cell lines enhanced the sensitivity of these cells to PTX significantly, where the IC_50_ value was reduced by 42% (BT#9-IC_50_ = 12.81 to PTX+BT#9 IC_50_ = 7.55; *p* < 0.0001) and 20% (BT#9-IC_50_ + 27.23 to PTX+BT#9 IC_50_ = 22.56; *p* < 0.0001) in HEY and OVCAR5 cell lines, indicating that a concurrent treatment of PTX and BT#9 may work synergistically in OC cells. These preliminary data need further investigation in other OC cell lines. However, it should be recognised that PTX also alters mitochondrial dynamics, biogenesis, and functions, which results in ROS production, leading to cellular toxicity [65,66,67].

In addition to the in vitro cytotoxicity studies in OC cell lines, we demonstrate that BT#9 treatment significantly reduced the HEY cell line-induced tumour burden in mice. Daily oral gavages of BT#9 significantly reduced the tumour burden and abrogated intraperitoneal tumour metastasis in mice compared to the control untreated and captisol-treated mice. In addition, long-term suppression of functional Magmas by daily oral gavages of BT#9 resulted in a prolonged survival period in mice. Furthermore, the fact that treatment with BT#9 oral gavages on its own has no harmful effect on mouse organs such as the liver, pancreas, and small intestine suggests that targeting mitochondrial Magmas by BT#9 may be an effective approach for the treatment of OC patients, with potentially minimal harmful effects on other organs.

## 5. Conclusions

Platinum and taxane-based combination chemotherapy is a standard of care following debulking surgery in OC patients. However, almost all OC patients eventually experience a relapse due to failure of chemotherapy treatments, resulting in premature death. Hence, treatment for OC patients represents a major challenge, and the identification of new effective therapies is crucial for this group of patients.

This study highlights that the expression of Magmas is essential for the sustenance of OC progression against extensive ROS production experienced by the OC cells in the hostile TME. The increased expression of Magmas in high grades/stages of OC provides an adaptive response for tumour cells by neutralising ROS levels to promote tumour growth and function. This makes Magmas a potential target for therapeutic intervention. This study, for the first time, showed that not only increased ROS levels produced in response to BT#9 treatment are necessary for effective cancer cell killing but other pathways may also contribute to the damaging effects of BT#9. Normal cells are comparatively resistant to BT#9-induced cell killing compared to OC cell lines, and BT#9 treatment does not involve unwanted side effects on abdominal organs such as the liver, pancreas, and small intestine, as other cancer treatments necessitate. Hence, therapeutic intervention with BT#9 may present an improved method of treating OC patients without the need for additional auxiliary treatments prerequisite for standard chemotherapy treatments in patients. In addition, our previous data have shown that BT#9 has greater cytotoxicity towards carboplatin-resistant OC cell lines compared to non-resistant parental cell lines [41], further supporting that the development of Magmas-based therapeutics may have a strong potential for better treatment outcomes not only in newly diagnosed patients but also in platinum-resistant OC patients who currently do not have many treatment options.

**Figure 14:**
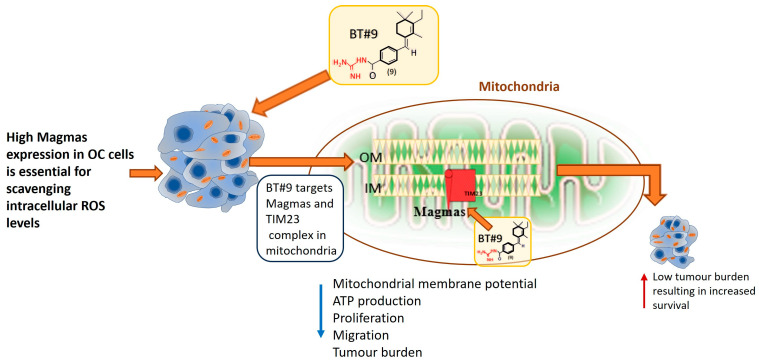
High expression of Magmas sustains OC progression by compromising ROS treatment experienced by the OC cells in the hostile tumour microenvironment. However, the increased ROS levels produced by BT#9 treatment effectively kill OC cells but spare normal cells, indicating BT#9 has an efficacious therapeutic potential. OM: outer membrane; IM: inner membrane.

Figure 14 recapitulates the mechanism of action of BT#9 treatment in OC cells encapsulating the mouse xenograft.

## Figures and Tables

**Table 1:** Description of ovarian cancer cell lines used in this study.

Cell Line	Origin	Growth Medium Used
HEY (ovarian papillary cystadenocarcinoma, peritoneal deposit) (Buick, Pullano, and Trent 1985) [50]	High-grade ovarian serous adenocarcinoma	RPMI + 10% FBS + 1% Penstrep
OVCAR5 (high-grade ovarian adenocarcinoma, derived from ascites, chemo-naïve) (Fogh and Trempe 1975 [51]; Langdon and Lawrie 2001) [52]	High-grade ovarian serous adenocarcinoma	RPMI + 10% FBS + 1% Penstrep
A2780 (sparse information on cell line, obtained from untreated primary tumour material, https://www.culturecollections.org.uk/nop/product/a2780) (accessed on 10 September 2024)	Ovarian endometrioid adenocarcinoma	DMEM + 10% FBS + 1% Penstrep
OAW28 (obtained from the ascites of a patient with ovarian cystadenocarcinoma, https://www.culturecollections.org.uk/nop/product/oaw28) (accessed on 10 September 2024)	High-grade ovarian serous adenocarcinoma	DMEM + 10% FBS + 1% Penstrep
SKOV3 (derived from the ascitic fluid of a patient with adenocarcinoma, https://www.culturecollections.org.uk/nop/product/sk-ov-3) (accessed on 10 September 2024)	Ovarian serous cystadenocarcinoma	RPMI + 10% FBS + 1% Penstrep
OV90 (epithelial-like cell isolated from an ovary of a patient with malignant papillary serous adenocarcinoma, https://www.atcc.org/products/crl-3585) (accessed on 10 September 2024)	Ovarian adenocarcinoma	RPMI + 10% FBS + 1% Penstrep
COV318 (epithelial serous carcinoma cell line obtained from peritoneal ascites, https://www.culturecollections.org.uk/nop/product/cov318) (accessed on 10 September 2024)	High-grade ovarian serous adenocarcinoma	DMEM + 10% FBS + 1% Penstrep
COV504 (human ovarian epithelial serous carcinoma cell line established from pleural effusion, https://www.culturecollections.org.uk/nop/product/cov504) (accessed on 10 September 2024)	Ovarian carcinoma	DMEM + 10% FBS + 1% Penstrep
COV362 (human epithelial endometrioid carcinoma cell line established from pleural effusion, https://www.culturecollections.org.uk/nop/product/cov362) (accessed on 10 September 2024)	High-grade ovarian serous adenocarcinoma	DMEM + 10% FBS + 1% Penstrep
OVCAR3 (epithelial cells obtained from malignant ascites of a patient with progressive adenocarcinoma of an ovary, https://www.atcc.org/products/htb-161) (accessed on 10 September 2024)	High-grade ovarian serous adenocarcinoma	DMEM + 10% FBS + 1% Penstrep
ES2 (human clear cell carcinoma isolated from the ovary of a patient with progressive clear cell carcinoma, https://www.atcc.org/products/crl-1978#) (accessed on 10 September 2024)	High-grade ovarian clear cell carcinoma	RPMI + 10% FBS + 1% Penstrep
FT282 (obtained from a normal fallopian tube of a donor, https://www.atcc.org/products/crl-3449) (accessed on 10 September 2024)	Normal fallopian tube cell line	DMEM-HAM’S F12 + 2% USG and 1% Penstrep

## Data Availability

The datasets presented in the manuscript are part of a PhD thesis. If required, the data can be obtained from the corresponding author or Federation University, Australia, Mount Helen Campus library, upon request.

## Supplementary Materials

The following supporting information can be downloaded at https://www.mdpi.com/article/10.3390/cells14090655/s1, Table S1: Description of ovarian tumours used in this study; Figure S1: Expression of Magmas in OC cell lines. Protein expression of Magmas was evaluated in HEY, OVCAR5, and normal fallopian tube FT282 cell lines by IF using anti-Magmas mouse monoclonal antibody as described in Methods; Figure S2: (A) Expression of the Magmas in ES2 cell line. (B) IC_50_ value of BT#9 in the ES2 cell line. (C) Effect of varying Log10 concentrations of captisol on the viability of the ES2 cell line; Figure S3: Effect of BT#9 on the apoptotic and necrotic response of the OVCAR5 cell line; Figure S4: Brightfield images of the representative HEY cell line from one experiment treated with IC_25_ and IC_50_ values of BT#9 and stained with (A) caspase 3/7 and (B) PI as described in Figure 6; Figure S5: Effect of BT#9 on ROS production in OVCAR5 cells using the DCFDA cellular ROS detection assay kit, according to the manufacturer’s instructions, as described for HEY cells in Figure 8; Figure S6: Effect of BT#9 on the cellular bioenergetics of the HEY cell line; Figure S7: Representative live images of control untreated, captisol-treated, and BT#9 oral gavaged mice on day 28 of the experiment; Figure S8: mRNA expression of Magmas in BT#9 (n = 4), captisol (CAP, n = 5), and control (PBS, n = 5) xenografts.

## Abbreviations

The following abbreviations are used in this manuscript:

OC	Ovarian cancer
ROS	Reactive oxygen species
4-HNE	4-Hydroxynonenol
PI	Propidium iodide
DCFDA	Dichlorodihydrofluorescein diacetate
TCA	Tricarboxylic cycle
FIGO	International Federation of Obstetrics and Gynaecology
HGOSC	High-grade serous ovarian carcinoma
PI3K	Phosphatidylinositol 3 kinase
TME	Tumour microenvironment
GM-CSF	Granulocyte macrophage colony-stimulating factor
PAM	Pre-sequence translocated-associated motor subunit
TIM	Translocase of the inner membrane subunit
mthsp70	Mitochondrial heat shock protein 70
ETC	Electron transport chain
ACTH	Adrenocorticotrophic hormone
PKC	Protein kinase C
GH/PRL	Growth hormone/prolactin
HUVECs	Human umbilical vein endothelial cells
SOD	Superoxide dismutase
FDA	Food and Drug Administration
RWH	Royal Women’s Hospital
VCB	Victorian Cancer Biobank
DAB	3,3′-diaminobenzidine
IHC	Immunohistochemistry
H&E	Hematoxylin and eosin
IF	Immunofluorescence
PBST	Phosphate-buffered saline with Tween 20
BSA	Bovine serum albumin
DAPI	4′,6-diamidino-2-phenylindole
SDS-PAGE	Sodium dodecyl-sulphate polyacrylamide gel electrophoresis
PVDF	Polyvinylidene difluoride
HRP	Horse radish peroxidase
RNS	Reactive nitrogen species
TBHP	Tert-butyl hydrogen peroxide
PTX	Paclitaxel
NHMRC	National Health and Medical Research Council
TEAB	Triethyl ammonium bicarbonate
EACR	Extracellular acidification rate
OCR	Oxygen consumption rate
OXPHOS	Mitochondrial oxidative phosphorylation
