# Supplementary material for: Enhanced Expression of Mitochondrial Magmas Protein in Ovarian Carcinomas: Magmas Inhibition Facilitates Antitumour Effects, Signifying a Novel Approach for Ovarian Cancer Treatment
Source: Cells. 2025 Apr 29;14(9):655. doi: 10.3390/cells14090655 (PMC12071367; doi:10.3390/cells14090655)
Supplement: Supplementary file 1 [file cells-14-00655-s001.zip › cells-3558560-supplementary.pdf]

Supplementary Materials

Supplemental Table S1. Description of ovarian tumours used in the study.

Block #	Age of Patient	Pathologist's Diagnosis	Tumour Type	FIGO Stage	Silverberg classification	CA125 levels	Genetic History	Survival after diagnosis
2	20	Benign sclerosis tumour	Benign	--	--	Increased	Nil Ca	--
4	43	Benign serous cystadenoma	Benign	--	--	N/A	Peutz-Jegher's syndrome	--
5	54	Fibroma mitotically active	Benign	--	--	N/A	Nil Ca	--
6	64	Serous cystadenofibroma	Benign	--	--	N/A	Family history of Ca	--
7	58	Serous cystadenofibroma	Benign	--	--	N/A	N/A	--
8	62	Serous cystadenofibroma	Benign	--	--	N/A	N/A	--
9	48	Simple serous cyst	Benign	--	--	N/A	HNPCC carrier (colorectal Ca)	--
10	54	Serous cystadenoma borderline	Borderline	Ia	Not graded	N/A	Nil Ca	--
11	60	Serous borderline tumour	Borderline	Ia	Not graded	raised	Nil Ca	--
12	37	Serous cystadenoma-borderline, microinvasion	malignant	Ia	Not graded	N/A	No (grandfather Ca Pancreas)	--
13	49	Serous borderline tumour	Borderline	Ib	Not graded	raised	Nil Ca	--
15	31	Micropapillary serous carcinoma	malignant	Ic	G1	N/A	Grandmother gastric Ca, grandfather smoker lung Ca	--
16	67	Serous borderline tumour	Borderline	Ic	Not graded	N/A	niece and nephew bowel Ca	--
17	44	Serous borderline tumour	Borderline	Ic	Not graded	369	N/A	--
18	54	Serous carcinoma	malignant	IIc	G3	300	Mother kidney Ca, Br Ca	--
19	67	Serous carcinoma	malignant	IIc	G3	N/A	Sister Died melanoma	--
20	61	Serous carcinoma	malignant	IIc	G3	109	Nil Ca	--
21	72	Serous carcinoma†	malignant	IIc	G3	N/A	Nil Ca	--
22	65	Serous carcinoma	malignant	IIb	G2	1404	Nil Ca	--
23	74	Serous carcinoma†	malignant	IIIa	G3	N/A	BRCA2+ve	--
24	37	Micropapillary Serous carcinoma	malignant	IIIc	G1	N/A	No (Nil father liver Ca-drinker)	--

25	45	Serous carcinoma	malignant	IIIc	G1	N/A	N/A	--
26	71	Serous carcinoma	malignant	IIIc	G2	N/A	Nil Ca	--
27	42	Serous carcinoma	malignant	IIIc	G2	1200	Family history of Ca	--
28	43	Serous carcinoma	malignant	IIIc	G2	N/A	Nil Ca	3 years 7 months
29	57	Serous carcinoma	malignant	IIIc	G3	raised	Nil Ca	--
30	76	Serous carcinoma	malignant	IIIc	G3	N/A	NIL Ca	4 years 1 months
31	54	Serous carcinoma	malignant	IIIc	G3	1230	BRCA2+ve	--
32	62	Serous carcinoma	malignant	IIIc	G3	N/A	BRCA1+ve	2 years 7 months
33	56	Serous carcinoma	malignant	IIIc	G3	Increased	-ve, Nil Ca	2 years 5 months
34	38	Serous carcinoma	malignant	IIIc	G3	Increased	Nil Ca	--
35	60	Serous carcinoma	malignant	IIIc	G3	374	BRCA2 +ve	4 years 7 months
36	59	Serous carcinoma	malignant	IV	G2	N/A	sister with Ca	6 years 10 months
37	61	Serous carcinoma	malignant	IV	G2	raised	BRCA2 carrier	3 years 11 months

†Primary site is Fallopian tube; -ve= negative; +ve= positive; Ca = cancer; N/A = not available; G1 = grade 1; G2 = grade 2; G3 = grade 3

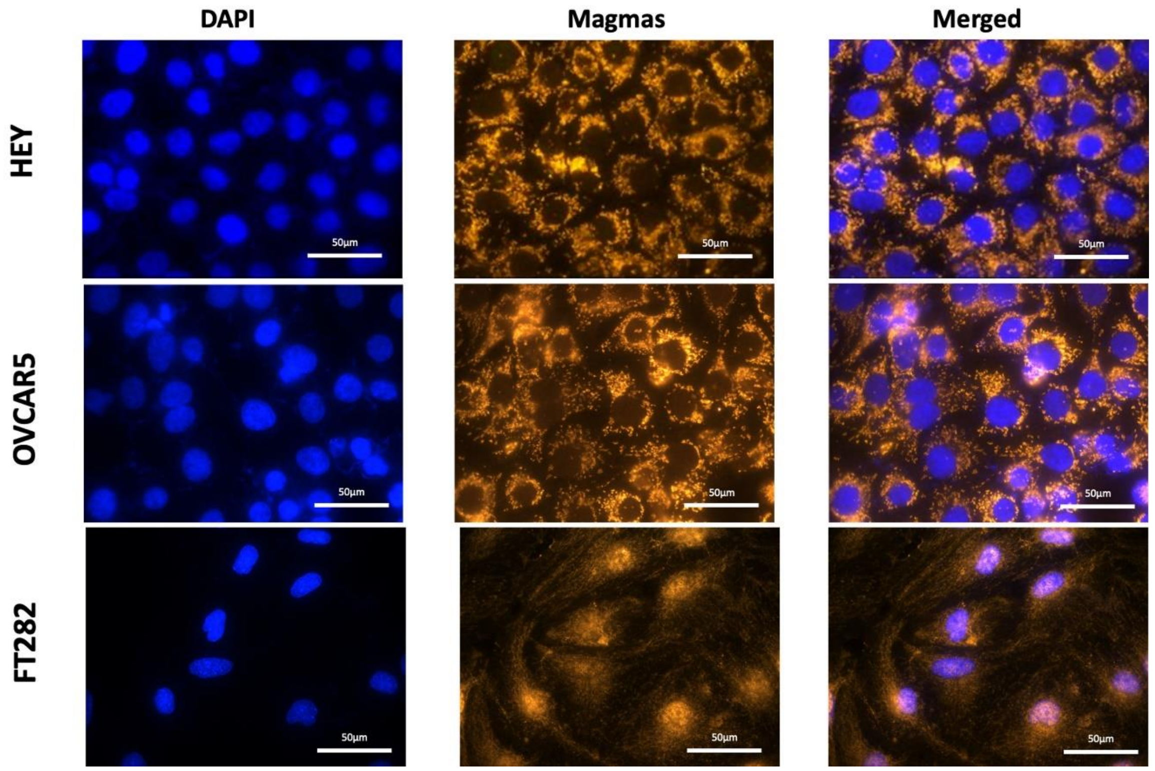

Supplemental Figure S1. Expression of Magmas in OC cell lines. Protein expression of Magmas was evaluated in HEY, OVCAR5 and normal fallopian

tube derived FT282 cell lines by IF using anti Magmas mouse monoclonal antibody as described in the Methods. Staining was visualized using the secondary anti-mouse Alexa 555 (orange) fluorescent-labelled antibody and nuclei were detected by DAPI (blue) staining. 20X magnification; scale bar: 50 µm.

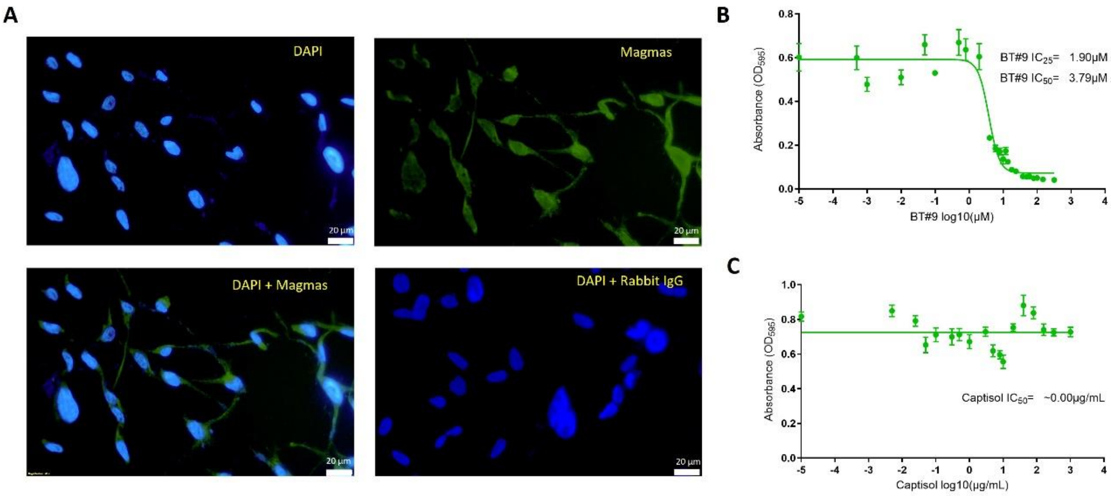

Supplemental Figure S2. (A) Expression of Magmas in ES2 cell line. Protein expression of Magmas was evaluated by IF using anti Magmas mouse monoclonal antibody as described in the Methods. Staining was visualized using the secondary goat anti-rabbit Alexa 488 (green) fluorescent-labelled antibody; nuclei were detected by DAPI (blue) staining; combined DAPI and Magmas staining; rabbit IgG as a primary control. 20X magnification; scale bar: 20 µm. (B) IC_{50} value of BT#9 in ES2 cell line. Briefly, ES2 cell line was treated with varying Log10 concentrations of BT#9. Cell viability was obtained by the MTT assay. Data are representative of three independent experiments using cells at different passages treated in triplicate. (C) Effect of varying Log10 concentration of captisol on the viability of ES2 cell line. ES2 cell line was treated with varying Log10 concentrations of captisol. Cell viability was obtained by the MTT assay. Data are representative of three independent experiments using cells at different passages treated in triplicate.

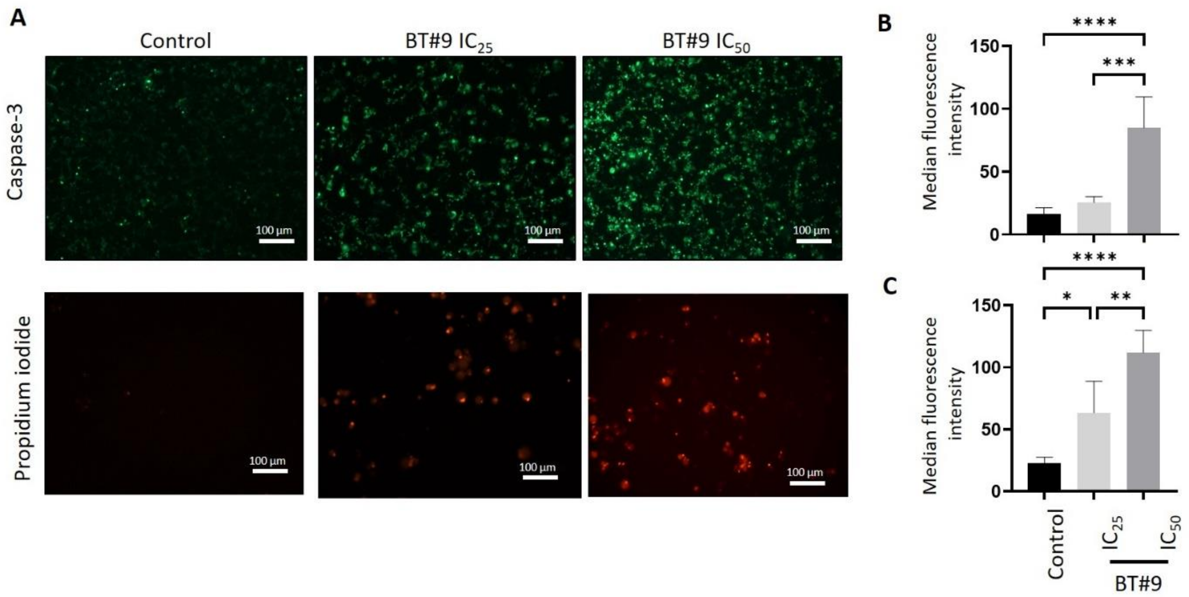

Supplemental Figure S3. Effect of BT#9 on the apoptotic and necrotic response of OVCAR5 cell line. (A) Immunofluorescence images of active caspase 3/7 and PI stained OVCAR5 cells was captured after treatment with IC_{25} and IC_{50} values of BT#9 for 12 hrs as described in the Methods. Images represent three independent experiments done in triplicate. Magnification 20X, scale bar 100 µm. (B) Bar graphs represent median fluorescence intensity of caspase 3/7 and PI staining at the respective BT#9 concentrations, for n=3 experiments done in triplicate. (C) The mitochondrial SOD level in the OVCAR5 cell line treated with IC_{25} and IC_{50} BT#9 concentrations was determined by the SOD assay kit, as described in Methods. The experiment was repeated three times, and data are presented as n = 3. Values are mean ±SD with significance deduced by using One-way ANOVA (Tukey's multiple comparison test) and indicated by ****$p<0.0001$, ***$p<0.001$, **$p<0.01$ and *$p <0.05$.

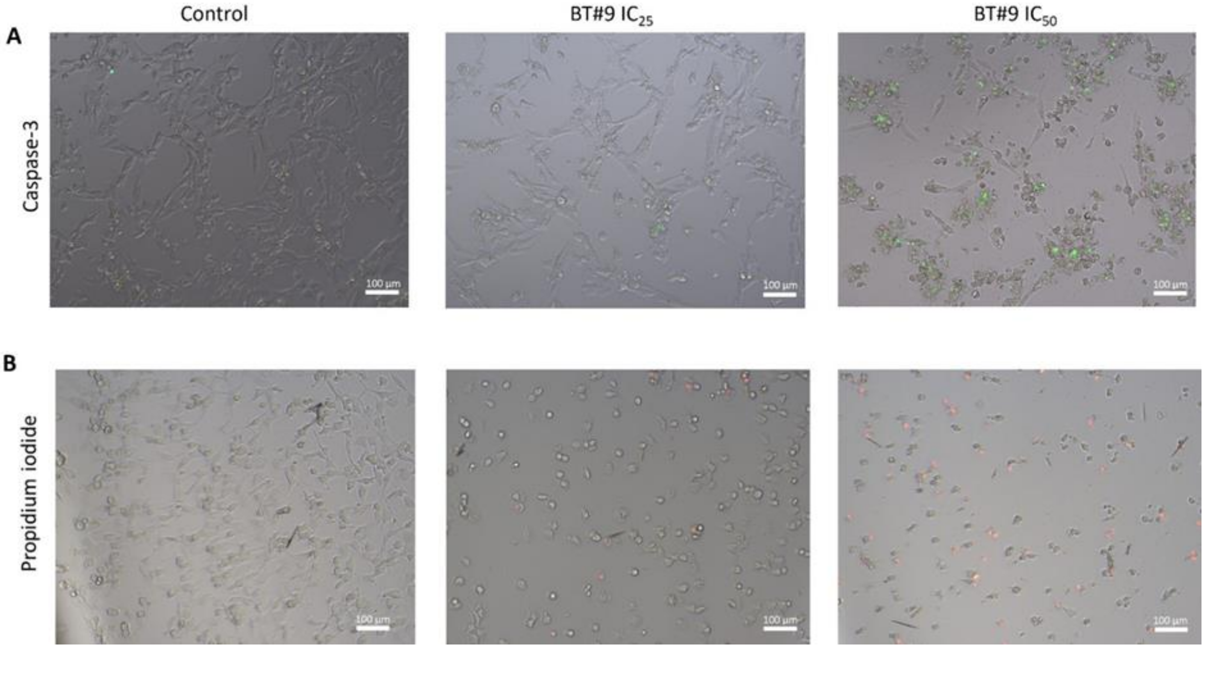

Supplemental Figure S4. Bright field images of representative HEY cell line from one experiment treated with IC_{25} and IC_{50} values of BT#9 and stained with (A) caspase 3/7 and with (B) PI as described in Figure 6. Magnification 20X, scale bar 100 µm.

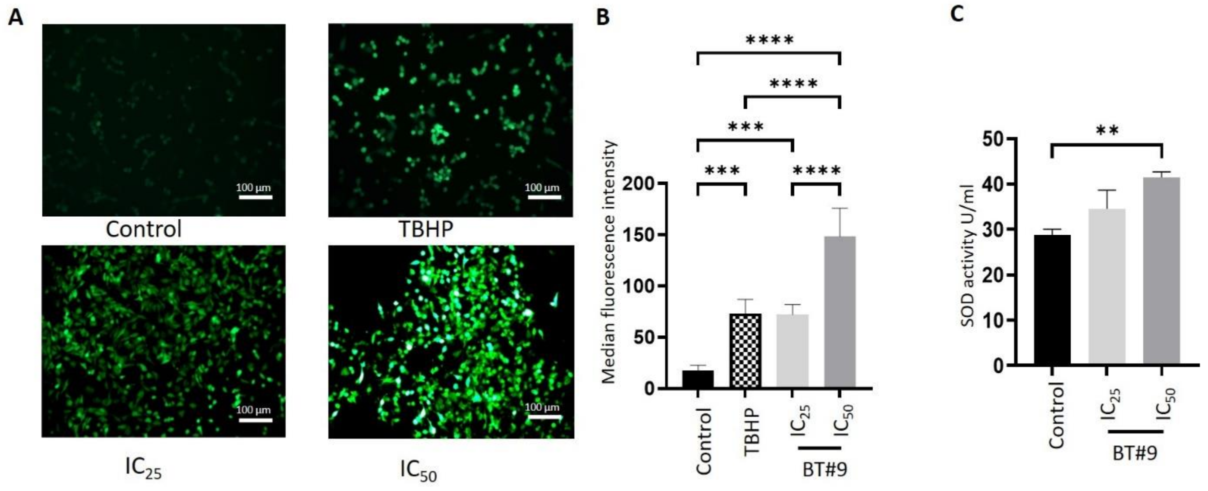

Figure S5. Effect of BT#9 on ROS production in OVCAR5 cells using the DCFDA cellular ROS detection assay kit according to the manufacturer's instructions as described for HEY cells in Figure 8. (A) OVCAR5 cell line was treated with IC_{25} and IC_{50} concentrations of BT#9. ROS generation was observed under a fluorescence microscope at 20X magnification scale bar 100 µm. (B) Bar graphs represent median fluorescence intensity of DCFDA. Values are mean ±SD with significance deduced by using One-way ANOVA (Tukey's multiple comparisons test) and indicated by ****$p<0.0001$, ***$p<0.001$ when compared to untreated cells. Tert-butyl hydrogen peroxide (TBHP) acts as a positive control and mimics ROS activity to oxidize DCFDA to fluorescent DCF. (C) Mitochondrial SOD level in OVCAR5 cell line treated with IC_{25} and IC_{50} BT#9 concentrations was determined by SOD assay kit as described in Methods. The experiment was repeated three times and data is presented as n =3. Values are mean ±SD with significance deduced by using One-way ANOVA (Dunnett's multiple comparisons test) and indicated by **$p<0.01$ when compared to untreated cells.

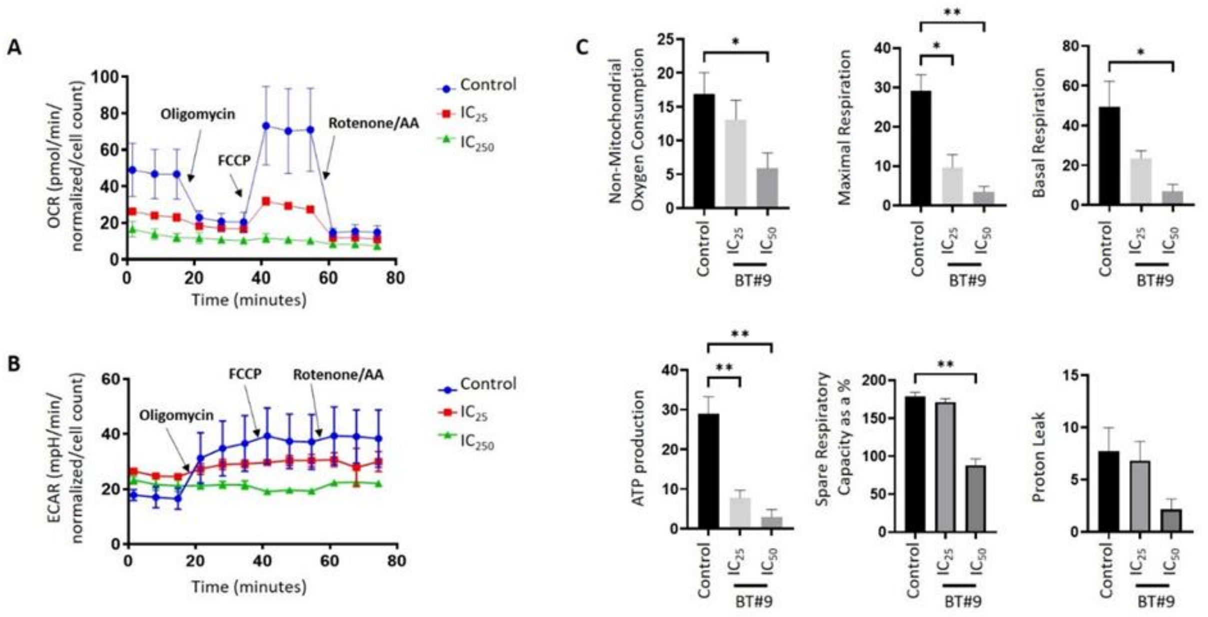

Supplemental Figure S6. Effect of BT#9 on the cellular bioenergetics of HEY cell line. Cellular bioenergetics was evaluated by Mito Stress Assay in response to treatments with IC_{25} and IC_{50} values of BT#9 for 12 hrs. Representative graph of measurements of (A) oxygen consumption rate (OCR), (B) extracellular acidification rate (ECAR) following sequential addition of oligomycin (ETC complex V inhibitor, inhibits ATP synthase), FCCP (inhibitor of mitochondrial membrane potential) and Rotenone (ETC complex 1 inhibitor) as described in Methods. (C) Bar graphs represent non-mitochondrial oxygen consumption rate, maximal respiration, basal respiration, ATP production, spare respiratory capacity and proton leak measured by using the parameters described in (A). Each parameter was normalized to total protein concentration. The experiment was repeated three times and data is presented as n = 3; values are mean ± SD; with significance deduced by using One-way ANOVA (Dunnett's multiple comparison test) and indicated by $**p<0.01$, $*p<0.05$ when compared to HEY untreated cells.

Control Captisol BT#9

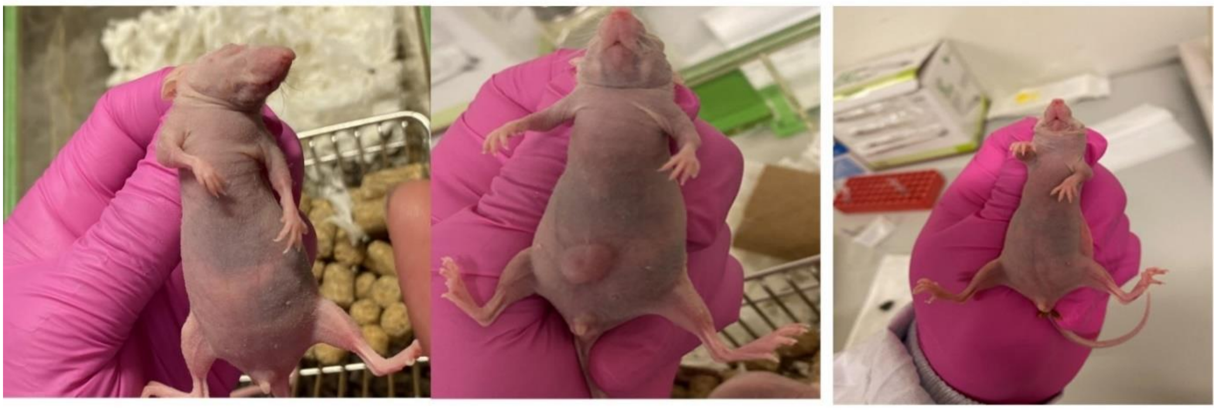

Supplemental Figure S7. Representative live images of control untreated, captisol treated and BT#9 oral gavaged mice at day 28 of the experiment.

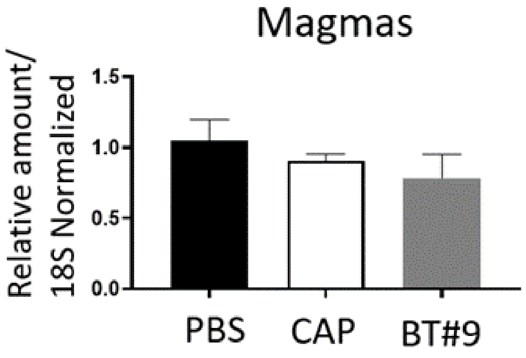

Supplemental Figure S8. mRNA expression of Magmas in BT#9 (n=4), Captisol (CAP, n=5) and control (PBS, n=5) xenografts.
